# Optimization of a non-activating medium for short-term chilled storage of barramundi (*Lates calcarifer*) testicular spermatozoa

**DOI:** 10.1007/s10695-023-01191-8

**Published:** 2023-05-17

**Authors:** Adrien F. Marc, Jarrod L. Guppy, Hayley Marshall, Dean R. Jerry, Donna Rudd, Damien B. B. P. Paris

**Affiliations:** 1 Gamete and Embryology (GAME) Laboratory, College of Public Health, Medical & Veterinary Sciences, James Cook University, Townsville, QLD 4811 Australia; 2 College of Public Health, Medical, and Veterinary Sciences, James Cook University, Townsville, QLD 4811 Australia; 3 Centre for Sustainable Tropical Fisheries and Aquaculture, College of Science and Engineering, James Cook University, Townsville, QLD 4811 Australia; 4 Australian Research Council Industrial Transformation Research Hub for Advanced Prawn Breeding, James Cook University, Townsville, QLD 4811 Australia; 5 Tropical Futures Institute, James Cook University, Geylang, Singapore; 6 Australian Institute of Tropical Health and Medicine, James Cook University, Townsville, QLD 4811 Australia

**Keywords:** Fish, Extender, Biochemical, Computer-assisted sperm analysis, Flow cytometry, Sperm quality

## Abstract

Reliable short-term chilled sperm storage is a critical prerequisite to using advanced reproductive techniques for captive breeding of barramundi (Asian sea bass; *Lates calcarifer*). Marine Ringer's solution (MRS) is a common non-activating medium (NAM) and has previously been used to store sperm from wild-caught barramundi. However, MRS-stored spermatozoa from captive-bred barramundi were observed to lyse within 30 min incubation. Therefore, this study aimed to optimize the composition of NAM for short-term chilled storage by characterizing and mimicking the biochemical profile of seminal and blood plasma of captive-bred barramundi. To further understand the effect of each component, osmolality was first examined to determine its effect on sperm viability. Thereafter, the effects of NaHCO_3_, pH, and Na^+^ and K^+^ concentrations on sperm motility were investigated. Optimization of the NAM formula was achieved through iterative adaptions. The increase in NAM osmolality from 260 to 400 mOsm/kg led to a significant improvement in sperm viability. Moreover, using HEPES instead of NaHCO_3_ as buffering agent significantly enhanced sperm motility and velocity. As a result, sperm samples diluted with optimized NAM (185 mM NaCl, 5.1 mM KCl, 1.6 mM CaCl_2_·2H_2_O, 1.1 mM MgSO_4_·7H_2_O, 10.0 mM HEPES, 5.6 mM D^+^ glucose, 400 mOsm/kg, pH 7.4) and stored at 4 °C showed no significant loss in total motility for up to 48 h and retained progressive motility for up to 72 h. The optimized NAM developed in this study significantly extended the functional lifespan of spermatozoa during chilled storage, permitting the ongoing development of advanced reproductive technologies for barramundi.

**Supplementary Information:**

The online version contains supplementary material available at 10.1007/s10695-023-01191-8.

## Introduction

Barramundi (Asian sea bass; *Lates calcarifer*) is an aquaculture species of commercial importance farmed throughout Australia, Southeast Asia, and increasingly globally (Moore 1979). Over the last decade, the increasing domestic demand for Australian-farmed barramundi has outstripped production (Savage 2016). Thus, the Australian barramundi aquaculture industry has set the ambitious goal of meeting market demand by 2025 and increasing annual production from 9,000 to 25,000 tonnes (FRDC 2020). One key driver to achieving this goal is to implement selective breeding programs to enhance traits of commercial interest. However, the operational efficiency of current selective breeding programs is hindered by complications associated with barramundi's (1) mass-spawning reproductive strategy (i.e., skewed parental contribution, necessitating DNA testing, etc.) (Robinson et al. 2010; Domingos et al. 2013, 2014, 2021) and (2) ability to sex change (i.e., barramundi are protandrous hermaphrodites) (Moore 1979; Budd et al. 2015, 2022b; Banh et al. 2017; Domingos et al. 2018; Budd 2020). Therefore, a priority for the barramundi industry is to achieve better control over reproduction to boost the efficiency of selective breeding programs (Robinson and Jerry 2009).

Methods to induce sex change in barramundi from male to female are now available, which will increase the efficiency of selective breeding programs by increasing the number of young broodstock females available for spawning (Banh et al. 2021a, b; Budd et al. 2022a; Guppy et al. 2022). Yet, unsynchronized gonadal maturation still occurs in males and females reared in captivity, skewing parental contribution in mass-spawning events (Frost et al. 2006; Robinson et al. 2010; Domingos et al. 2013, 2014, 2021; Loughnan et al. 2013) and preventing the reliable collection of mature spermatozoa and eggs for artificial fertilization (Marc et al. 2021; Guppy et al. 2022). Developing a reliable short-term sperm storage protocol could overcome these problems and allow temporary access to spermatozoa of barramundi broodstock (Contreras et al. 2020). Sperm storage coupled with refinement of egg-stripping and artificial fertilization will enhance control over mate pairings and, therefore, improve the effectiveness of barramundi breeding programs (Robinson et al. 2010; Domingos et al. 2013, 2014, 2021; Jerry et al. 2022).

Various non-activating media (NAM) have been used in teleosts for short-term sperm storage, including Stop-milt, Cortland, Ringer's solution, Mountib medium, and seminal-like solutions (Contreras et al. 2020). These NAM are used to ease sperm handling and enhance storage duration and are designed as a substitute for seminal plasma (Billard and Cosson 1992; Cabrita et al. 2010; Beirao et al. 2019; Contreras et al. 2020). In teleosts, seminal plasma provides spermatozoa the ability to activate and sustain motility once released from the genital tract into the external aqueous environment (Billard and Cosson 1992; Beirao et al. 2019; Cosson 2019; Dietrich et al. 2021). Seminal plasma also creates an optimal environment in the spermatic duct for spermatozoa to be stored immotile until the spawning event (Billard and Cosson 1992; Beirao et al. 2019; Cosson 2019; Dietrich et al. 2021). The seminal plasma composition differs among species (Ulloa-Rodríguez et al. 2017). For instance, the seminal plasma osmolality of teleosts ranges from 200 to 400 mOsm/kg, excluding sturgeons (i.e., 50 to 100 mOsm/kg) (Alavi and Cosson 2006; Alavi et al. 2011) and the seminal plasma pH ranges between 7.5 and 8.5 (Alavi and Cosson 2005; Islam and Akhter 2012; Cosson 2019; Contreras et al. 2020). Moreover, Na^+^, K^+^, Ca^2+^, and Mg^2+^ are the main mineral compounds in most seminal plasma, which play a role at species-specific concentrations in sperm motility-activating signaling (Cabrita et al. 2010; Beirao et al. 2019; Cosson 2019). Therefore, it is necessary to ensure the formulation of NAM is suitable for the species of interest because an ionic imbalance of NAM during chilled storage could alter sperm function or cause premature activation of motility (Dreanno et al. 1999; Alavi et al. 2004; Yang et al. 2006; Bozkurt et al. 2008; Jing et al. 2009).

Short-term chilled sperm storage (Leung 1987; Palmer et al. 1993; Vuthiphandchai et al. 2021) and artificial fertilization (Palmer et al. 1993; Haque et al. 2021; Vuthiphandchai et al. 2021) have been achieved using sperm collected from wild-caught barramundi. These studies used Ringer's solution as a base NAM (Leung 1987; Palmer et al. 1993; Vuthiphandchai et al. 2021). However, spermatozoa collected from captive-bred broodstock swelled and lysed within 30 min of incubation in marine Ringer's solution, requiring an osmolality increase from 260 to 400 mOsm/kg to prevent osmotic stress from occurring (Marc et al. 2021). Although sperm viability improved sufficiently to enable sperm quality assessment, total sperm motility remained low (mean: 24.5 ± 4.4%) (Marc et al. 2021). While low motility could be indicative of poor sperm quality in broodstock, it is necessary to ensure that the Ringer's-based NAM used to dilute sperm provides optimal conditions for triggering and maintaining sperm motility. Therefore, further investigations were required to determine the suitability of Ringer's-based NAM for handling and short-term chilled storage of captive-bred barramundi spermatozoa.

This study investigated: (i) the biochemical properties of barramundi seminal and blood plasma to gain a greater understanding of the microenvironment to which testicular spermatozoa are exposed in the sperm duct, and the general internal body physiology of captive broodstock reared according to the Australian aquaculture industry standards; (ii) the influence of the Ringer's-based NAM composition, including the effect of osmolality on sperm viability, and the effects of pH, Na^+^ and K^+^ concentrations, and NaHCO_3_ *vs.* HEPES buffering agents on sperm motility; and (iii) the ability of the optimized NAM, modified based on outcomes of (i) and (ii), to retain sperm motility after short-term chilled storage.

## Methods

### Animals

Captive-bred barramundi were maintained at the Marine and Aquaculture Research Facility (MARF), James Cook University, Townsville, Queensland, Australia. Two cohorts of male barramundi were used in this study: (i) broodstock (*n* = 8) for blood plasma (mean body weight 3.5 ± 0.2 kg, length 62.0 ± 1.4 cm, maintained in 2,500 L tanks at 28 °C, 30 ppt salinity, and 16 h light: 8 h dark cycle), and (ii) broodstock (*n* = 10) for seminal plasma and spermatozoa (mean body weight 3.9 ± 0.6 kg, length 70.1 ± 4.8 cm, maintained in 28,000 L tanks under standard breeding conditions at 30 °C, 30 ppt salinity, and 16 h light: 8 h dark cycle). Experiments operated under James Cook University Animal Ethics Permit (A2406).

### Sample collection and preparation

Blood was collected from the caudal vein of sedated fish using 18G 3.5" spinal needles (Provet Qld Pty Ltd, Brisbane, Australia). A total blood volume of 3 mL was extracted from each fish. Blood samples were centrifuged immediately after collection, and plasma samples were held on ice before storage at -80 ℃ until analysis. Sperm samples were collected following methods described by Marc et al. (2021). Broodstock were sedated in an anaesthetic bath containing 40 mg/L iso-eugenol (AQUI-S®, New Zealand), and the gonopore of males was rinsed with distilled water before sperm collection. Sperm samples were retrieved from the gonads by cannulation, and an aliquot was assessed for quality control (i.e., presence of blood, urine contamination, premature sperm activation, or failure to activate on contact with saltwater) before being deemed suitable for inclusion in subsequent experiments.

### Biochemical composition and osmolality

Blood and seminal plasma were analyzed for 12 biochemical parameters, including sodium (Na^+^), potassium (K^+^), calcium (Ca^2+^), magnesium (Mg^2+^), chlorine (Cl^−^), phosphate (PO_4_^3−^), partial pressure of carbon dioxide (pCO_2_), total protein, cholesterol, glucose, triglyceride, and urea, using an Olympus AU 480 Clinical Chemistry Analyzer (Beckman Coulter, USA). Osmolality was measured by a freezing point depression osmometer (Osmomat 030; Genotec GmbH, Berlin, Germany), and pH was recorded using a micro-pH electrode (inlab ultra-micro-ISM; Mettler‐Toledo, Melbourne, Victoria, Australia).

### Assessment of sperm function

#### Sperm motility

Sperm motility was assessed following methods described by Marc et al. (2021). Specifically, the computer-assisted sperm analysis (CASA) system consisted of an Olympus BX53/CoolLED pE-300W fluorescent phase-contrast microscope (Olympus, Tokyo, Japan) equipped with a 20 X negative phase-contrast objective, 0.5 X C-Mount adaptor, and a Basler avA1000-100gc high frame-rate area scan camera (Basler AG, Ahrensburg, Germany), coupled with AndroVision®, version 1.1 software (Minitüb GmbH, Tiefenbach, Germany). Videos of spermatozoa were recorded at 10 X final magnification.

Multiple sperm motility parameters were evaluated, including total motility (TM; %), progressive motility (PM; %), slow, medium, and fast motility (%), curvilinear velocity (VCL; μm/s), average path velocity (VAP; μm/s), straight-line velocity (VSL; μm/s), the amplitude of lateral head displacement (ALH; μm), straightness of the average path (STR; % of VSL/VAP), the linearity of the curvilinear path (LIN; % of VSL/VCL), wobble (WOB; % of VAP/VCL) and beat-cross frequency (BCF; Hz). Sperm motility categories were classified based on VCL thresholds, including total motility (VCL ≥ 15 μm/s), progressive motility (VCL ≥ 35 μm/s), slow motility (VCL ≥ 15 and < 35 μm/s), medium motility (VCL ≥ 35 and < 100 μm/s), and fast motility (VCL ≥ 100 μm/s).

Sperm motility assessment was conducted in triplicate for each fish and assessed 15 s after activation in natural saltwater (30 ppt) consisting of 404.9 mM Na^+^, 459.1 mM Cl^−^, 10.1 mM K^+^, 9.7 mM Ca^2+^, 0.1 mM PO_4_^3−^, pH 8.1, and 900 mOsm/kg using a two-step dilution to a final ratio of 1:1000 (1:10 in NAM and 1:100 in saltwater). Leja 4-chamber linear-flow slides (Leja Products B. V., Nieuw Vennep, Amsterdam, Netherlands) pre-warmed at 30 ˚C were used to contain sperm samples during analysis. Sperm motility was performed on 1 s videos recorded at a frame rate of 60 fps.

#### Sperm viability

Sperm viability was assessed following methods described by Marc et al. (2021) using Hoechst 33,342/Propidium Iodine (PI) dual nuclear stains and flow cytometry with 20 mW, 488 nm; 27.5 mW, 635 nm; and 27 mW, 405 nm lasers (CyanADP flow cytometer, Beckman Coulter, Fullerton, CA, USA). Both FSC and SSC were set to Lin, and FL3 (PE-Texas Red) and FL6 (DAPI) were set to Log in the Detector/Amps window. Detector voltages were set to: FSC = 400 V, SSC = 600 V, FL6 = 750 V, and FL3 = 780 V.

The concentration of each sperm sample was standardized to 100 × 10^6^ sperm/mL. An aliquot from each sample was used to generate an unstained control (U1), heat-treated Hoechst single-stained positive control (P1), heat-treated Hoechst/PI double-stained positive control (P2), and test sample. Positive controls were incubated at 70 °C for 5 min to induce perforation of the sperm plasma membrane and subsequent complete PI staining. Positive controls and test samples were each incubated at room temperature for 30 min in 10 μg/mL Hoechst 33,342 and incubated for 7 min in the dark in 10 μg/mL PI. Debris was excluded from the sperm population using side and forward scatter profiles from unstained samples. The viable sperm subpopulation was gated based on threshold quadrants set on unstained and stained positive controls. Flow cytometry data were analyzed using Summit version 4.4 software (Beckman Coulter, Fullerton, California, USA).

### Experimental design

Marine Ringer's solution (MRS), described by Palmer et al. (1993), was used as a starting medium for developing an optimized NAM. MRS consisted of 124.1 mM NaCl, 5.1 mM KCl, 1.6 mM CaCl_2_·2H_2_O, 1.1 mM MgSO_4_·7H_2_O, 0.1 mM NaHCO_3_, 2.6 mM NaH_2_PO_4_·2H_2_O, and 5.6 mM D^+^ glucose with a final osmolality of 260 mOsm/kg adjusted to pH 7.4 using 0.1 M NaOH. This study used an iterative experimental design to optimize the NAM to suit sperm samples collected from captive-bred barramundi reared in 30 ppt saltwater.

#### Experiment 1. Effect of NaHCO_3_-buffered NAM osmolality on sperm viability

In this experiment, sperm samples (*n* = 5) were collected from barramundi broodstock. Sperm volume was 37.4 ± 8.1 µL, sperm concentration was 29.8 ± 5.8 × 10^9^ sperm/mL, and total sperm count was 1,144.1 ± 285.6 × 10^6^ sperm. Each sperm sample was individually diluted 1:10 in 260 mOsm/kg NAM (MRS original osmolality control) and 300, 350, 400, and 450 mOsm/kg NAM treatments using NaCl. The range of osmolality of NaHCO_3_-buffered NAM was modified based on the osmolality of captive-bred barramundi seminal plasma determined in this study. Sperm viability analyses were performed after 1 h incubation at 4 ℃.

#### Experiment 2. Effect of NaHCO_3_-buffered NAM pH on sperm motility

For this experiment, the optimal NAM determined in Experiment 1 was selected, which consisted of 182.4 mM NaCl, 5.1 mM KCl, 1.6 mM CaCl_2_·2H_2_O, 1.1 mM MgSO_4_·7H_2_O, 0.1 mM NaHCO_3_, 2.6 mM NaH_2_PO_4_·2H_2_O, and 5.6 mM D^+^ glucose with a final osmolality of 400 mOsm/kg adjusted to the required pH level using 0.1 M NaOH. For the control, NAM was adjusted to pH 7.4 based on the original formula (Palmer et al. 1993). For the treatments, NAM was adjusted to pH 6.5 to determine if acidic conditions inhibited sperm motility (Chantzaropoulos et al. 2015), pH 7.8 based on the pH of barramundi blood plasma, pH 8.1 to replicate the pH of saltwater rearing tank, and pH 8.5 to mimic the pH of natural saltwater. Sperm samples from barramundi (*n* = 10) were collected. Sperm volume was 8.0 ± 1.3 µL, sperm concentration was 29.9 ± 3.9 × 10^9^ sperm/mL, and total sperm count was 245.1 ± 55.1 × 10^6^ sperm. Each sperm sample was individually incubated at 4 ℃ for 1 h and 24 h undiluted and diluted at 1:10 in the different NAM before sperm motility was assessed. Undiluted sperm samples were activated directly following 1:1000 dilution in 30 ppt saltwater and were used as a control to quantify the effect of NAM on sperm motility.

#### Experiment 3. Effect of HEPES-buffered NAM pH on sperm motility

Based on Experiment 2 and Supplementary Material 1 (Fig. S1 and Table S1), we hypothesized that the NaHCO_3_ buffering agent might be the element inhibiting sperm motility when diluted in NAM. Therefore, the pH experiment was repeated using a zwitterionic organic chemical buffering agent, HEPES, 4-(2-hydroxyethyl)-1-piperazineethanesulfonic acid, which has improved buffering stability at low temperatures compared to NaHCO_3_-based buffers (Williams-Smith et al. 1977; Matthews et al. 2018). Moreover, the monobasic sodium phosphate (NaH_2_PO_4_·2H_2_O) buffering agent was also removed from the NAM to prevent further unwanted interactions. For this experiment, NAM consisted of 182.4 mM NaCl, 5.1 mM KCl, 1.6 mM CaCl_2_·2H_2_O, 1.1 mM MgSO_4_·7H_2_O, 10.0 mM HEPES, and 5.6 mM D^+^ glucose with a final osmolality of 400 mOsm/kg adjusted to the required pH value using 0.1 M NaOH. Sperm samples from barramundi (*n* = 10) were collected. Sperm volume was 13.9 ± 3.4 µL, sperm concentration was 16.0 ± 2.5 × 10^9^ sperm/mL, and total sperm count was 239.0 ± 69.3 × 10^6^ sperm. Each sperm sample was individually incubated at 4 ℃ for 1 h and 24 h undiluted and diluted at 1:10 in the different NAM before sperm motility was assessed. Undiluted sperm samples were activated directly following 1:1000 dilution in saltwater and were used as a control to quantify the effect of NAM on sperm motility. In addition, based on the Experiment 2 outcome, the NaHCO_3_-buffered NAM adjusted at pH 6.5 was also used as a control.

#### Experiment 4. Effect of HEPES-buffered NAM sodium and potassium concentration on sperm motility

For this experiment, NAM consisted of NaCl and KCl, each adjusted to their required concentrations, 1.6 mM CaCl_2_·2H_2_O, 1.1 mM MgSO_4_·7H_2_O, 10.0 mM HEPES, and 5.6 mM D^+^ glucose with a final osmolality of 400 mOsm/kg adjusted to pH 7.4 using 0.1 M NaOH. Different treatments were prepared with the following NaCl/KCl concentrations: 0 mM NaCl/190 mM KCl, 140 mM NaCl/50 mM KCl, 160 mM NaCl/30 mM KCl, 185 mM NaCl/5 mM KCl, and 190 mM NaCl/0 mM KCl. NAM was adjusted to pH 7.4 with 1 M KOH for the 0 mM NaCl/190 mM KCl treatment and 0.1 M NaOH for the other groups. The first and last treatments, 0 mM NaCl/190 mM KCl and 190 mM NaCl/0 mM KCl, were designed to determine whether the motility of barramundi spermatozoa was Na^+^ or K^+^ dependent. The intermediate treatments spanned the breadth of seminal plasma ionic measurements. Sperm samples from barramundi (*n* = 6) were collected. Sperm volume was 14.2 ± 3.4 µL, sperm concentration was 19.0 ± 2.0 × 10^9^ sperm/mL, and total sperm count was 281.6 ± 73.9 × 10^6^ sperm. Each sperm sample was individually incubated at 4 ℃ for 1 h and 24 h undiluted and diluted at 1:10 in the different NAM before sperm motility was assessed. Undiluted sperm samples were activated directly following 1:1000 dilution in saltwater and were used as a control to quantify the effect of NAM on sperm motility.

#### Experiment 5. Evaluation of the optimized NAM for short-term chilled storage

This experiment compared the motility of sperm samples that were undiluted (control) or diluted in optimized NAM. Based on Experiments 1–4, optimized NAM consisted of 185 mM NaCl, 5.0 mM KCl, 1.6 mM CaCl_2_·2H_2_O, 1.1 mM MgSO_4_·7H_2_O, 10.0 mM HEPES, and 5.6 mM D^+^ glucose with a final osmolality of 400 mOsm/kg adjusted to pH 7.4 using 0.1 M NaOH. Sperm motility was assessed after 1, 24, 48, 72, and 96 h incubation at 4 ℃.

### Statistical analysis

Statistical analyses were performed using RStudio version 1.0.153 (RStudio, Inc, Boston, MA, USA). Percentage data for motility (TM, PM, Slow, Medium, and Fast), kinetic parameters (LIN, STR, and WOB), and viability were normalized using arcsine $\left(\sqrt{value/100}\right)$ transformation. All data were assessed for normality and homogeneity of variance using Shapiro–Wilk and Levene tests. Where data were normally distributed with homogeneous variance, one-way ANOVA, followed by Tukey's multiple comparison test, was used to test differences between treatments. A Kruskal–Wallis H test was performed when these assumptions were not met, followed by Fisher's Least Significant Difference test to compare treatment pairs. The paired samples Wilcoxon test (Wilcoxon signed-rank test) was used to evaluate significant differences between 1 and 24 h incubation for Experiments 2, 3, and 4. Kruskal–Wallis rank-sum test followed by pairwise Mann–Whitney U-test (Wilcoxon rank-sum test) with Bonferroni correction was used to assess the significance between groups at each time point for Experiment 5. Data are displayed as mean ± standard error (SEM). The level of significance was set at *P* < 0.05.

## Results

### Biochemical analysis

Ionic and metabolite concentrations of blood plasma were relatively homogenous between male barramundi. However, these parameters in seminal plasma varied substantially between individuals (Table 1). The concentration of Na^+^, Cl^−^, pCO_2_, glucose, triglyceride, and urea in blood and seminal plasma did not differ (Table 1). However, the concentration of K^+^ and PO_4_^3−^ were tenfold higher in seminal than blood plasma (Welch t-test, K^+^: *t*(7.02) = -8.74, *P* < 0.001; PO_4_^3−^: *t*(7.02) = -6.26, *P* < 0.001; Table 1). Mg^2+^ concentration was also significantly higher in seminal than blood plasma (Wilcoxon's test, *P* < 0.001, r = -0.89; Table 1). The higher ionic concentration in seminal plasma is consistent with an overall 18.8% increase in osmolality (mean: 396.1 ± 13.4 mOsm/kg) over blood plasma (mean: 333.5 ± 2.1 mOsm/kg; Table 1). By contrast, Ca^2+^, total protein, cholesterol, and pH were significantly lower in seminal than in blood plasma (Table 1).

**Table 1:** Ionic and metabolites compositions of seminal and blood plasma of captive barramundi (*Lates calcarifer*) broodstock reared in 30 ppt saltwater

Parameter	Blood plasma			Seminal plasma		
	Mean ± SEM	Range	CV	Mean ± SEM	Range	CV
Na^+^ (mM)	160.7 ± 0.9^a^ (8)	155.8 – 163.6	1.7	164.2 ± 5.1^a^ (8)	140.7 – 181.8	9.4
K^+^ (mM)	3.7 ± 0.1^a^ (8)	3.1 – 4.3	11.8	37.7 ± 3.6^b^ (8)	23.3 – 53.3	29.2
Ca^2+^ (mM)	2.6 ± 0.1^a^ (8)	2.4 – 3.0	6.9	1.5 ± 0.3^b^ (8)	0.5 – 2.9	59.4
Mg^2+^ (mM)	1.0 ± 0.0^a^ (8)	0.9 – 1.1	5.4	3.9 ± 1.1^b^ (8)	1.7 – 12.2	86.3
Cl^−^ (mM)	133.4 ± 1.0^a^ (8)	129.4 – 137.8	2.2	149.7 ± 7.2^a^ (8)	129.0 – 198.8	14.5
PO_4_ (mM)	1.5 ± 0.1^a^ (8)	1.1 – 1.8	16.4	15.4 ± 2.1^b^ (8)	7.9 – 27.3	40.7
pCO_2_ (mmHg)	2.6 ± 0.1^a^ (8)	2.4 – 3.0	6.3	5.6 ± 0.8^a^ (8)	2.0 – 10.0	44.5
TP (g.L^−1^)	44.3 ± 1.4^a^ (8)	36.4 – 49.6	9.7	28.5 ± 4.1^b^ (6)	14.4 – 46.0	38.8
CHO (mM)	5.6 ± 0.5^a^ (8)	4.2 – 8.2	25.8	1.7 ± 0.5^b^ (7)	0.0 – 3.0	83.0
Glu (mM)	3.7 ± 0.4^a^ (8)	2.3 – 5.9	33.0	2.1 ± 0.4^a^ (8)	0.4 – 3.9	60.7
TG (mM)	1.0 ± 0.2^a^ (8)	0.5 – 2.3	58.3	3.7 ± 1.9^a^ (4)	1.1 – 0.2	117.1
Urea (mM)	2.4 ± 0.1^a^ (8)	2.1 – 2.9	10.8	4.2 ± 0.8^a^ (3)	2.3 – 5.3	39.7
pH	7.8 ± 0.0^a^ (8)	7.7 – 7.9	0.9	7.5 ± 0.1^b^ (5)	7.2 – 7.8	3.6
Osm (mOsm/kg)	333.5 ± 2.1^a^ (8)	325 – 341	1.9	396.1 ± 13.4^b^ (7)	340 – 451	9.7

Data are mean ± standard error (SEM), number of fish (*n*), and coefficient of variation (CV) are reported for each parameter. Different letters indicate significant differences (*P* < 0.05) between blood and seminal plasma. Abbreviations: Total protein (TP), cholesterol (CHO), glucose (Glu), triglycerides (TG), and osmolality (Osm)

#### Experiment 1. Effect of NaHCO_3_-buffered NAM osmolality on sperm viability

**Fig. 1:**
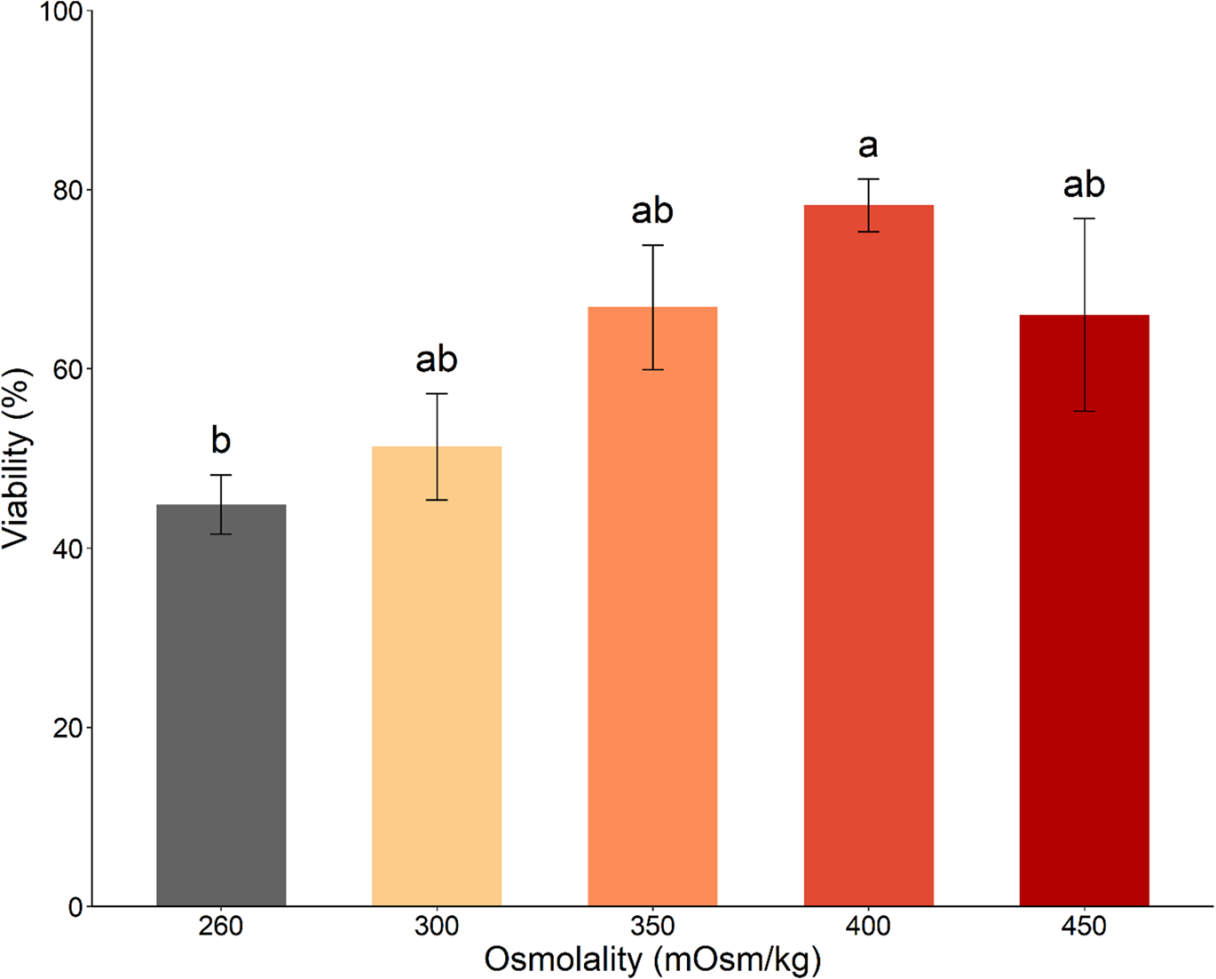
Effect of NaHCO_3_-buffered non-activating medium (NAM) osmolality on sperm viability. Percentage of live barramundi (*Lates calcarifer*) spermatozoa was analyzed using Hoechst 33,342/Propidium Iodine (PI) dual nuclear stains and flow cytometry 1 h post-incubation at 4 °C in NaHCO_3_-buffered NAM with an osmolality of 260 to 450 mOsm/kg (*n* = 5). Osmolality of NAM (Marine Ringer's solution consisted of 124.1 mM NaCl, 5.1 mM KCl, 1.6 mM CaCl_2_·2H_2_O, 1.1 mM MgSO_4_·7H_2_O, 0.1 mM NaHCO_3_, 2.6 mM NaH_2_PO_4_·2H_2_O, and 5.6 mM D^+^ glucose at pH 7.4 and osmolality 260 mOsm/kg) was modified using NaCl. Data are mean ± SEM. Different lowercase letters indicate a significant difference between treatments (*P* < 0.05)

The highest sperm viability rates occurred in NaHCO_3_-buffered NAM with osmolalities, closely mimicking the range of seminal plasma (350–450 mOsm/kg; Table 1; Fig. 1). In particular, viability was significantly higher after 1 h incubation in NaHCO_3_-buffered NAM adjusted to 400 mOsm/kg (mean: 78.2 ± 2.9%) when compared to the original NAM at 260 mOsm/kg (mean: 44.8 ± 3.3%; ANOVA, *F*(1, 23) = 11.18, *P* < 0.05; Fig. 1).

#### Experiment 2. Effect of NaHCO_3_-buffered NAM pH on sperm motility

**Fig. 2:**
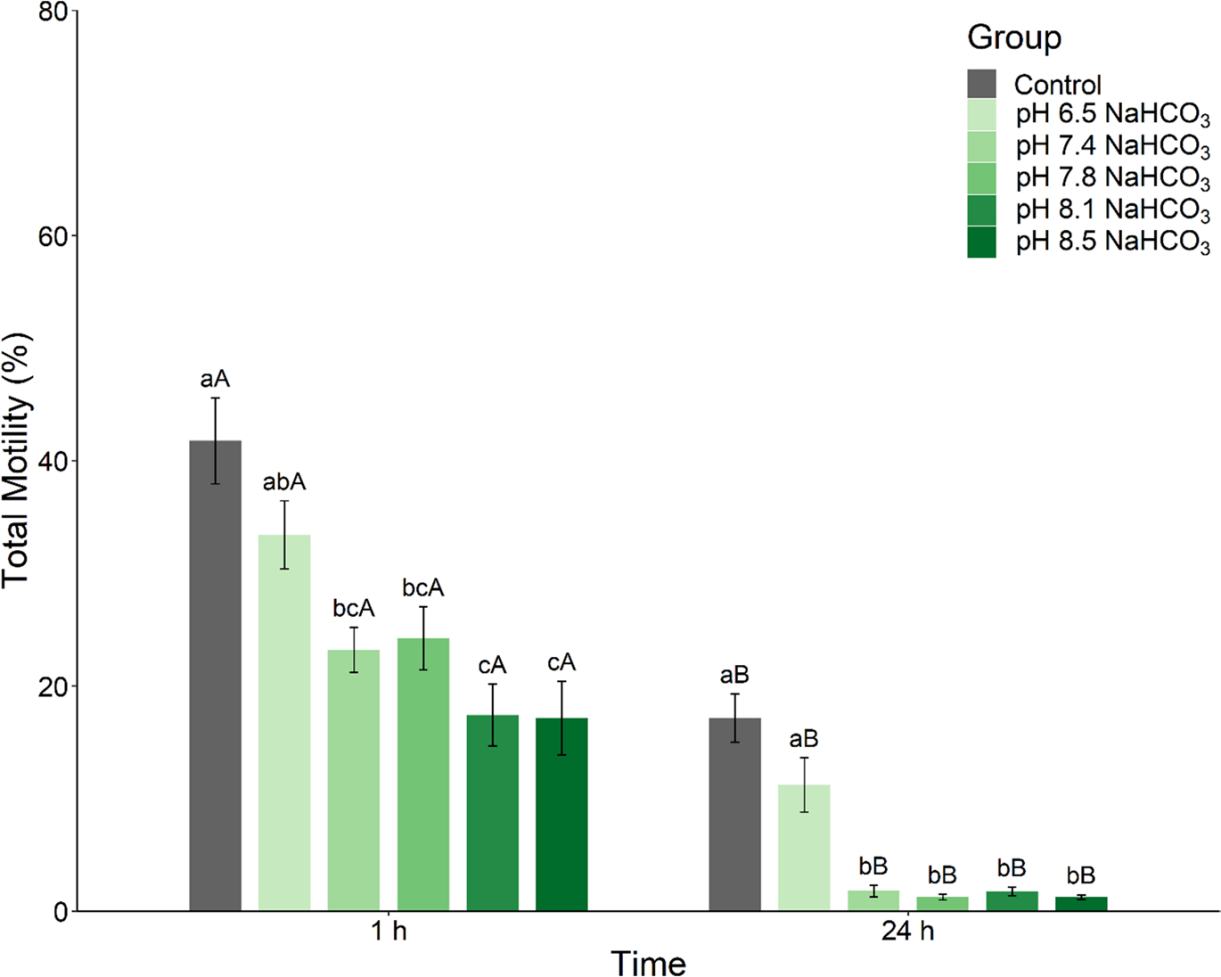
Effect of NaHCO_3_-buffered non-activating medium (NAM) pH on sperm motility. Total motility of barramundi (*Lates calcarifer*) spermatozoa was analyzed at 1 and 24 h post-incubation at 4 °C in NaHCO_3_-buffered NAM with different pH (*n* = 10). Undiluted sperm was used as a control. pH of NAM (Marine Ringer's solution consisted of 182.4 mM NaCl, 5.1 mM KCl, 1.6 mM CaCl_2_·2H_2_O, 1.1 mM MgSO_4_·7H_2_O, 0.1 mM NaHCO_3_, 2.6 mM NaH_2_PO_4_·2H_2_O, and 5.6 mM D^+^ glucose, osmolality 400 mOsm/kg) was modified using NaOH. Data are mean ± SEM. Different lowercase letters indicate a significant difference between pH treatments within an incubation period, and different capital letters indicate a significant difference between incubation periods at the same pH (*P* < 0.05)

After 1 h incubation, spermatozoa incubated in NaHCO_3_-buffered NAM with a pH adjusted between 7.4 and 8.5 showed a significant decline in TM compared to the control (ANOVA, *F*(5, 54) = 9.81, *P* < 0.01; Fig. 2). However, there were no significant differences in sperm swimming velocities (VCL, VSL, and VAP) between NaHCO_3_-buffered NAM treatments (Supplementary Table S2). After 24 h incubation, spermatozoa motility was decreased to less than 5% in NAM with pH adjusted between 7.4 and 8.5, while it was higher than 15% in control and NAM with pH of 6.5. For the same pH, TM was significantly decreased at 24 h post-incubation compared to 1 h post-incubation in NaHCO_3_-buffered NAM (Wilcoxon's test, Control: *P* = 0.002, r = 0.89; NAM pH 6.5: *P* = 0.0039, r = 0.854; Fig. 2; Supplementary Table S2). The motility of spermatozoa stored in NaHCO_3_-buffered NAM adjusted at pH 6.5 did not differ from the control at either incubation period (Fig. 2). Of note, biochemical analysis of NaHCO_3_-buffered NAM showed an increase of pCO_2_ with the rise in pH (pH 6.5: 0.0 mmHg; pH 7.4: 1.3 mmHg; pH 7.8: 2.4 mmHg; pH 8.1: 3.2 mmHg; pH 8.5: 4.5 mmHg).

#### Experiment 3. Effect of HEPES-buffered NAM pH on sperm motility

**Fig. 3:**
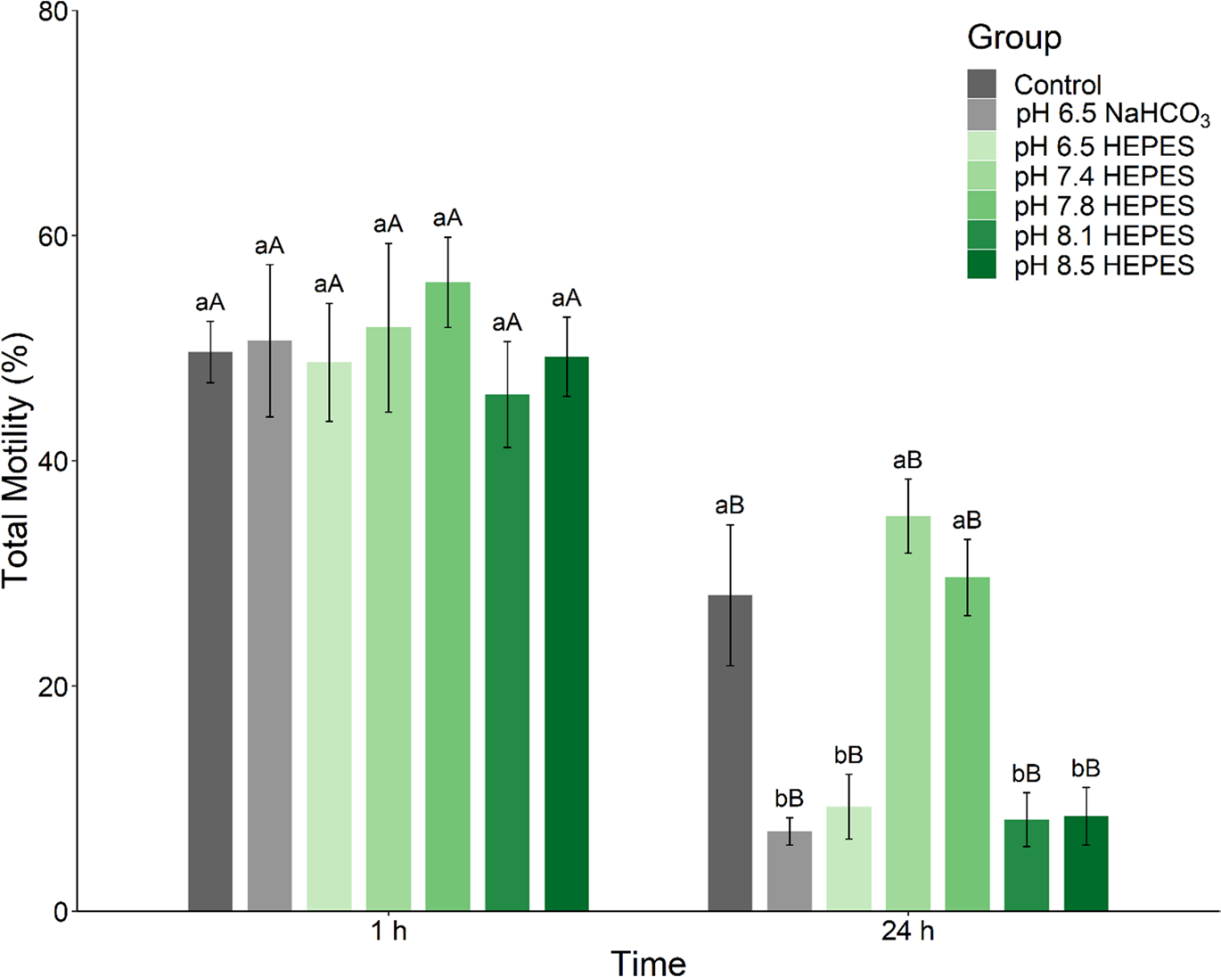
Effect of HEPES-buffered non-activating medium (NAM) pH on sperm motility. Total motility of barramundi (*Lates calcarifer*) spermatozoa was analyzed at 1 and 24 h post-incubation at 4 °C in HEPES-buffered NAM with different pH (*n* = 7). Undiluted sperm was used as a control as well as sperm diluted in the NaHCO_3_-buffered NAM pH 6.5 for comparison. pH of NAM (Marine Ringer's solution consisted of 182.4 mM NaCl, 5.1 mM KCl, 1.6 mM CaCl_2_·2H_2_O, 1.1 mM MgSO_4_·7H_2_O, 10.0 mM HEPES, and 5.6 mM D^+^ glucose, osmolality 400 mOsm/kg) was modified using NaOH. Data are mean ± SEM. Different lowercase letters indicate a significant difference between pH treatments within an incubation period, and different capital letters indicate a significant difference between incubation periods at the same pH (*P* < 0.05)

After 1 h incubation, sperm motility and velocity remained high, with no significant differences between NAM treatments (Fig. 3; Supplementary Table S3). Spermatozoa in the HEPES-buffered NAM adjusted at pH 7.8 showed the highest TM, followed by pH 7.4. For the same pH, TM was decreased at 24 h post-incubation compared to 1 h post-incubation (Wilcoxon's test*, P* = 0.031, r = 0.898; Fig. 3). However, HEPES-buffered NAM at pH 7.4 and pH 7.8 and the control retained higher TM than other treatments (ANOVA, *F*(6, 35) = 12.23, *P* < 0.001; Fig. 3; Supplementary Table S3). Spermatozoa held in HEPES-buffered NAM adjusted at pH 6.5, pH 8.1, and pH 8.5 showed a significant decline in sperm motilities (TM, PM, and Fast) and kinetics (VCL, VSL, VAP, and ALH) compared to pH 7.4 and 7.8 treatments (Supplementary Table S3). Except for BCF, there were no significant differences in sperm motility characteristics between spermatozoa held in NaHCO_3_- and HEPES-buffered NAM at pH 6.5 after 1 h and 24 h incubation (Fig. 3; Supplementary Table S3).

#### Experiment 4. Effect of HEPES-buffered NAM sodium and potassium concentration on sperm motility

**Fig. 4:**
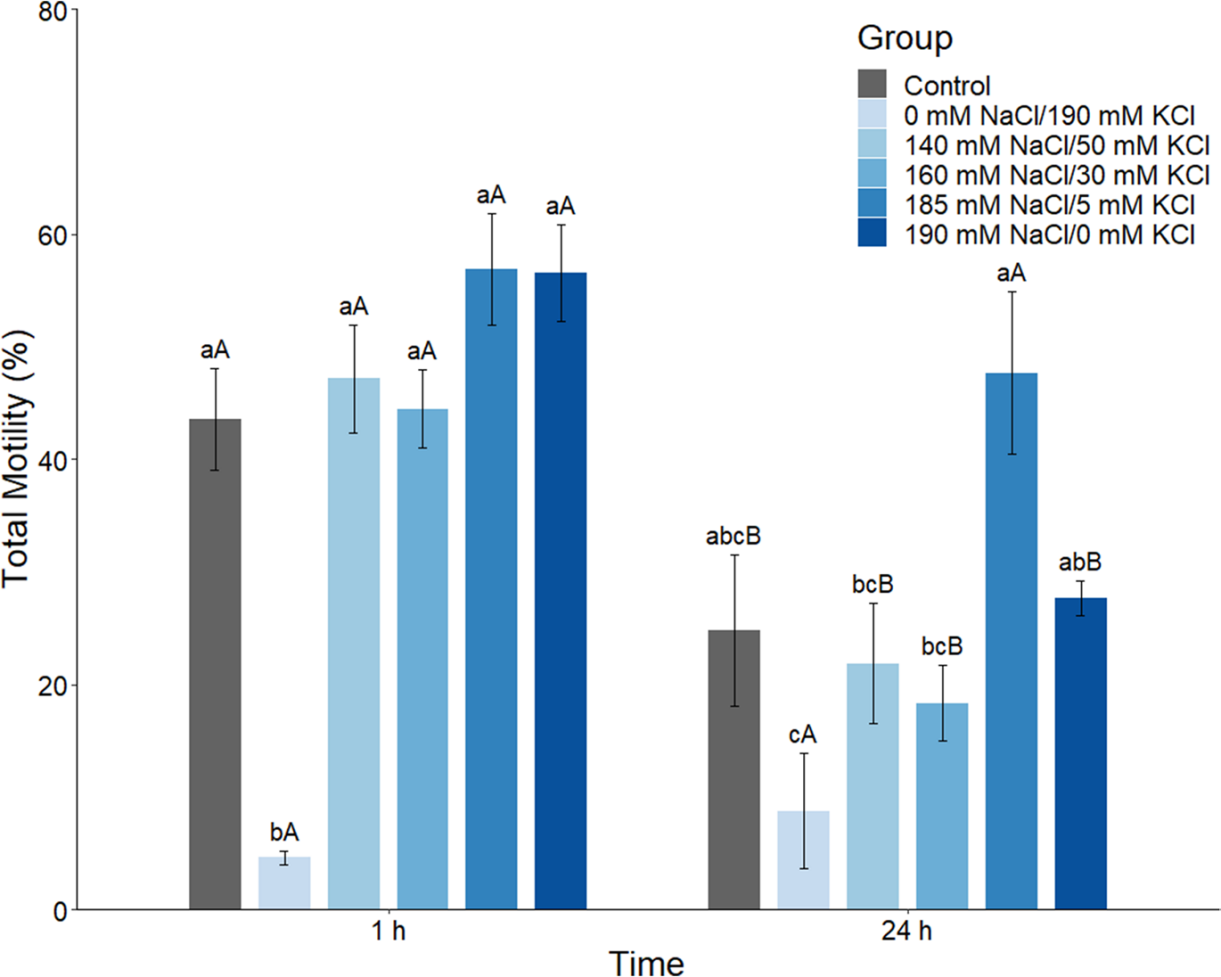
Effect of HEPES-buffered non-activating medium (NAM) NaCl and KCl concentration on sperm motility. Total motility of barramundi (*Lates calcarifer*) spermatozoa was analyzed at 1 and 24 h post-incubation at 4 °C in HEPES-buffered NAM with different NaCl and KCl concentrations (*n* = 6). Undiluted sperm was used as a control. NaCl and KCl concentrations of NAM (Marine Ringer's solution consisted of 0–190 mM NaCl, 0–190 mM KCl, 1.6 mM CaCl_2_·2H_2_O, 1.1 mM MgSO_4_·7H_2_O, 10.0 mM HEPES, and 5.6 mM D^+^ glucose at pH 7.4 and osmolality 400 mOsm/kg) were modified accordingly to maintain osmolality of 400 mOsm/kg. Data are mean ± SEM. Different lowercase letters indicate a significant difference between concentrations within an incubation period, and different capital letters indicate a significant difference between incubation periods at the same concentration (*P* < 0.05)

After 1 h incubation, spermatozoa incubated in Na^+^ free HEPES-buffered NAM showed a large decline in TM (mean: 4.6 ± 0.6%) compared to the undiluted sperm (control) and all other treatments (mean: 43.5 ± 4.5%; ANOVA, *F*(5, 30) = 20.88, *P* < 0.001; Fig. 4; Supplementary Table S4). By contrast, the K^+^ free (0 mM) and low K^+^ (5 mM) media with the highest Na^+^ concentrations had the highest TM, although these did not differ from control or Na^+^-containing HEPES-buffered NAM (mean: 56.5 ± 4.3% and 56.8 ± 5.0% respectively; Fig. 4; Supplementary Table S4). A similar trend was observed for PM, Fast, VCL, VSL, and VAP (Supplementary Table S4). In particular, the 185/5 mM NaCl/KCl medium showed the highest PM (mean: 28.5 ± 2.4%), with most spermatozoa classified as Fast (mean: 26.4 ± 2.3%) and having high overall sperm velocities (VCL: 112.1 ± 7.4 μm/s, VSL: 79.1 ± 6.8 μm/s, and VAP: 102.6 ± 7.6 μm/s). For all media containing Na^+^, the swimming trajectory of spermatozoa (LIN, STR, WOB, ALH, and BCF) indicated little deviation from the primary path (Supplementary Table S4). After 24 h incubation, the highest TM (mean: 47.7 ± 7.2%) was also recorded in the NAM containing 185 mM NaCl/5 mM KCl, although this did not differ from the control or NAM containing 190 mM NaCl. Furthermore, there was no significant difference between TM measured after 1 h and 24 h incubation in NAM containing 185 mM NaCl/5 mM KCl (Wilcoxon's test, *P* = 0.313, r = 0.469; Fig. 4; Supplementary Table S4). Of note, this experiment was initially conducted using the NaHCO_3_-buffered NAM at pH 7.4, 400 mOsm/kg (Supplementary Material 1, Fig. S1, and Table S1). However, the influence of NaCl and KCl concentration on sperm motility was masked due to the presence of NaHCO_3_ (Figs. 2 and 3; Supplementary Material 1, Fig. S1, and Table S1).

#### Experiment 5. Evaluation of the optimized HEPES-buffered NAM for short-term chilled storage

**Fig. 5:**
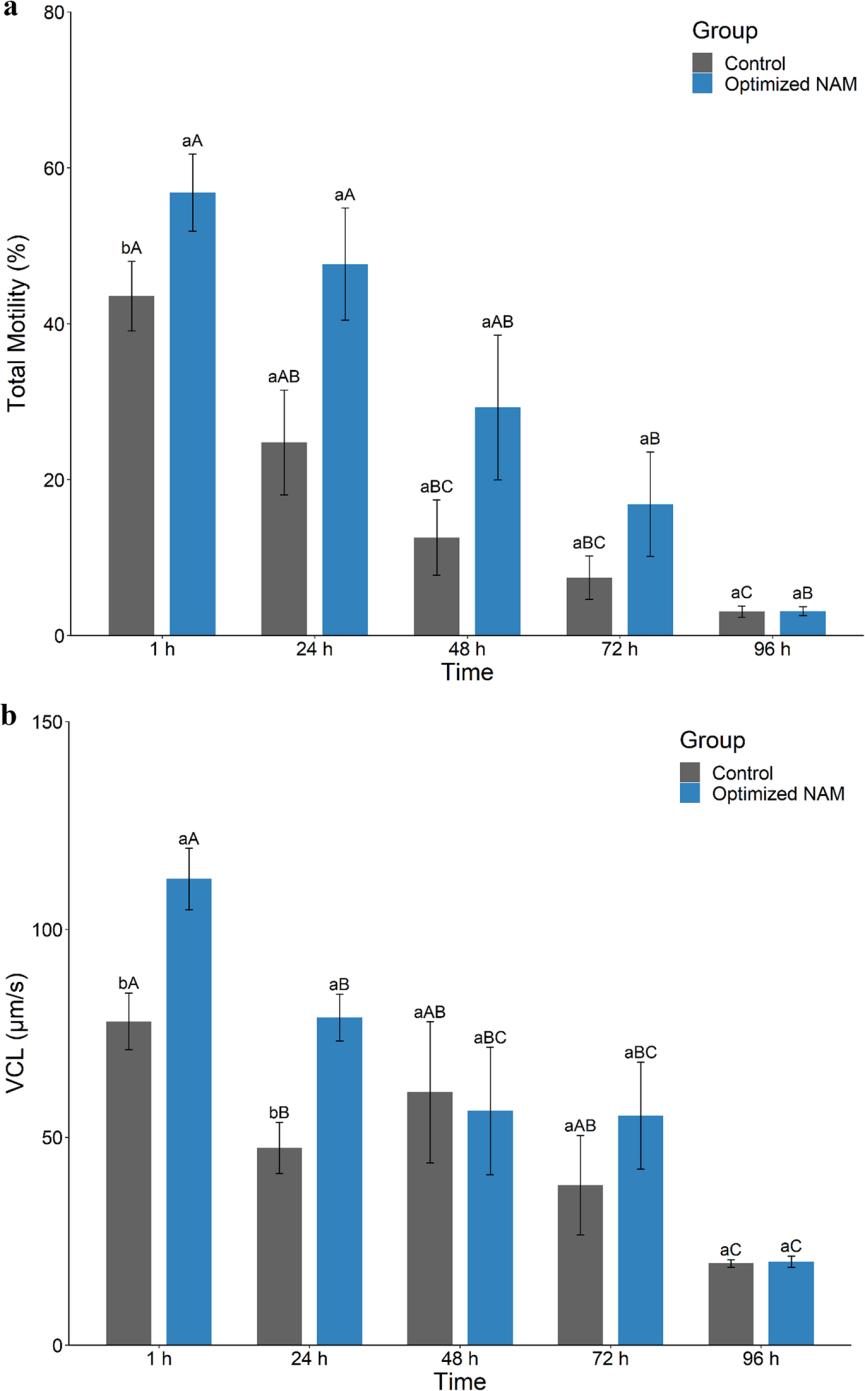
Evaluation of the optimized NAM post-short-term chilled storage on sperm motility. (a) Total motility and (b) VCL of barramundi (*Lates calcarifer*) spermatozoa were analyzed for up to 96 h at 4 °C in an optimized HEPES-buffered non-activating medium (*n* = 6). Undiluted sperm was used as a control. Optimized NAM consisted of 185 mM NaCl, 5.0 mM KCl, 1.6 mM CaCl_2_·2H_2_O, 1.1 mM MgSO_4_·7H_2_O, 10 mM HEPES and 5.6 mM D^+^ glucose at pH 7.4 and osmolality 400 mOsm/kg. Data are mean ± SEM. Different lowercase letters indicate a significant difference between extender treatments within an incubation period, and different capital letters indicate a significant difference between incubation periods for the same medium (*P* < 0.05)

After 1 h incubation, spermatozoa in the optimized HEPES-buffered NAM showed significantly higher motilities (TM, PM, and Fast) and velocities (VCL, VSL, VAP, LIN, and WOB) than those in the undiluted sperm control (Fig. 5a and b; Supplementary Table S5). Other swimming parameters (STR, ALH, and BCF) did not differ between treatments (Supplementary Table S5). TM only declined significantly after 72 h in the optimized HEPES-buffered NAM (Kruskal–Wallis Rank Sum test; H = 17.54, df = 4, *P* < 0.002) compared to 48 h for the undiluted sperm control (Kruskal–Wallis Rank Sum test; H = 18.45, df = 4, *P* = 0.001; Fig. 5a; Supplementary Table S5). However, VCL significantly declined within 24 h in the optimized HEPES buffered NAM (Kruskal–Wallis Rank Sum test; H = 18.43, df = 4, *P* = 0.001; Fig. 5b; Supplementary Table S5). Of note, for both time points, the decreases in average motility and velocity were mainly caused by the loss of sperm motility in samples from two out of six fish at 48 h and three out of six fish at 72 h rather than a ubiquitous drop in motility and velocity from all six fish (Supplementary Table S6). When samples that failed activation were excluded, TM was 42.6 ± 6.0% at 48 h and 31.6 ± 2.2% at 72 h, respectively (Supplementary Table S6). After incubation of spermatozoa for 96 h, TM was less than 5%, and PM was absent for both groups (Fig. 5a; Supplementary Table S5).

## Discussion

Reliable sperm handling and storage protocols are required to establish artificial breeding techniques (e.g., artificial fertilization and sperm banking) for the barramundi aquaculture industry. Previously published sperm handling and storage protocols were developed using sperm samples from wild-caught barramundi (Leung 1987; Palmer et al. 1993; Vuthiphandchai et al. 2021). However, these methods used Ringer's-based NAM and results could not be replicated using sperm samples collected from captive-reared broodstock. Therefore, to provide fundamental knowledge toward optimizing a suitable NAM for captive-bred barramundi, the ionic and metabolite profile of seminal plasma was characterized for the first time. Furthermore, this study investigated the effects of osmolality, pH, Na^+^ and K^+^ concentrations, and NaHCO_3_ *vs.* HEPES buffering agents on sperm motility. As a result, it was confirmed that an osmotic imbalance in previously used marine Ringer's solution caused spermatozoa to lyse, and the presence of NaHCO_3_ buffering agent inhibited sperm motility. Further, it was demonstrated that spermatozoa incubated in an optimized NAM (185 mM NaCl, 5.1 mM KCl, 1.6 mM CaCl_2_·2H_2_O, 1.1 mM MgSO_4_·7H_2_O, 10.0 mM HEPES, and 5.6 mM D^+^ glucose, 400 mOsm/kg, pH 7.4) could be stored at 4 ℃ for up to 48 h without significant loss of total motility.

Characterizing the biochemical parameters of blood and seminal plasma provided valuable insight into determining the root causes inducing sperm damage when stored in Ringer's-based NAM. The MRS used by Palmer et al. (1993) contained 124.1 mM of Na^+^ and had an osmolality of 260 mOsm/kg. The MRS osmolality was lower than seminal (mean: 396.1 ± 13.4 mOsm/kg) and blood plasma (mean: 333.5 ± 2.1 mOsm/kg) derived from captive-bred barramundi reared in 30 ppt saltwater. However, MRS sodium content and osmolality were similar to levels found in the blood plasma of barramundi reared in freshwater (Na^+^: 105.6 ± 2.8 mM and 218.3 ± 3.3 mOsm/kg; Supplementary Table S7) and seminal plasma of individuals reared in brackish water (15–17 ppt; osmolality range: 295 ± 4 to 306 ± 4 mOsm/kg) (Vuthiphandchai et al. 2021).

Palmer et al. (1993) previously used MRS as a base NAM for a cryopreservation experiment using sperm samples from wild-caught barramundi. Sperm samples incubated in MRS without cryoprotectant were not assessed for motility. However, they were used as a control group for the artificial fertilization experiment, resulting in an ~ 81% hatching rate (Palmer et al. 1993), confirming that the MRS successfully maintained barramundi sperm integrity during chilled storage. These findings directly contrasted our attempts to utilize MRS with spermatozoa collected from captive-bred barramundi reared in 30 ppt. In comparison, Palmer et al. (1993) caught barramundi in a coastal area, with the GPS coordinates of the fishing sites suggesting broodstock were captured in the freshwater plume or brackish section of the estuary. In addition, the seminal plasma osmolality was not assessed by Palmer et al. (1993) during the published experiment. However, Vuthiphandchai et al. (2021) found that the seminal plasma osmolality of barramundi reared in brackish conditions (15–17 ppt) ranged from 295 to 306 mOsm/kg, which differs from our findings (mean: 396.1 ± 13.4 mOsm/kg). While the effect of environmental salinity on seminal plasma osmolality remains to be determined in barramundi, an increase in seminal plasma osmolality with the increase in rearing salinity has been shown in tilapia, species which also has a high tolerance to a wide range of environmental salinity (Harvey and Kelley 1984; Linhart et al. 1999; Legendre et al. 2016). The seminal plasma of tilapia (*Sarotherodon melanotheron heudelotii*) reared in freshwater (0 ppt) was an average of 318 mOsm/kg compared to 330 mOsm/kg at 35 ppt and 349 mOsm/kg at 70 ppt (Legendre et al. 2016). A similar result was found in another tilapia species (*Oreochromis mossambicus*), where the osmolality of testicular seminal plasma increased by 15 mOsm/kg from 336.63 mOsm/kg to 351.0 mOsm/kg in fish reared in saltwater compared to freshwater (Linhart et al. 1999). Moreover, tilapia (*O. mossambicus*) transferred from freshwater to seawater showed an increase of seminal plasma osmolality from 283 mOsm/kg to 330 mOsm/kg after 30–90 days acclimation (Harvey and Kelley 1984). Consequently, we hypothesized that (1) the difference in environmental salinity is a driver of the discrepancy between Palmer et al. (1993) and our study, and (2) the optimal NAM osmolality is contingent on the rearing environment.

However, additional factors could be at the origin of the difference in seminal plasma osmolality and, thus, the suitability of the NAM; it is well documented that seminal plasma characteristics fluctuate within species. For example, in Atlantic cod (*Gadus morhua*), seminal plasma composition can differ significantly across spawning seasons and origin (wild-caught *vs.* captive-bred individuals) (Butts et al. 2010; Anthony et al. 2011). Hulak et al. (2008) also recorded a drop in seminal plasma osmolality when Northern pike (*Esox Lucius*) sperm samples were collected using manual stripping (mean: 273 ± 21 mOsm/kg) compared to cannulation (mean: 358 ± 77 mOsm/kg). Therefore, it will be of interest to investigate the influence of environmental salinity and collection methods (cannulation *vs.* manual stripping) on barramundi spermatozoa and seminal plasma to improve the chilled storage procedure.

In this study, we found that the NaHCO_3_ buffering agent in the NAM inhibited barramundi sperm motility. Specifically, NaHCO_3_ buffer caused a significant decline in motility at pH 7.4 or greater but not pH 6.5. The only discernible difference between the NAM at pH 6.5 and NAM at pH ≥ 7.4 was the presence of pCO_2_ in NAM at pH ≥ 7.4. It is known that NaHCO_3_ dissociates in an aqueous solution into CO_2_ + H_2_CO_3_ (free CO_2_), HCO_3_^−^, and CO_3_^2−^, with the equilibrium ratio of each chemical species present being pH-dependent (Burt and Rau 1994). At pH 7.4 and above, free-CO_2_ transmutes into HCO_3_^−^ and H^+^, whereas at pH 6.5, free-CO_2_ remains a gas at equilibrium. These results suggest three possible scenarios. Firstly, barramundi spermatozoa could take up HCO_3_^−^ into the cytoplasm through osmosis using Cl^−^/HCO_3_^−^ and Na^+^/HCO_3_^−^ exchanger proteins similar to mammals (Nishigaki et al. 2014). Thus, an increase in internal HCO_3_^−^ concentration will cause an intracellular accumulation of H^+^ through the K^+^ channel, leading to a drop in internal pH levels limiting further H^+^ uptake necessary for the initiation of motility (Tanaka et al. 2002; Inaba et al. 2003). Secondly, like in the Japanese eel, the internal increase in HCO_3_^−^ and H^+^ could be mediated by the conversion of CO_2_ through carbonic anhydrase, limiting further H^+^ uptake (Tanaka et al. 2002). Alternatively, like in turbot (*Scophthalmus maximus*), sperm motility could be inhibited by HCO_3_^−^ through its direct action on the flagellum axoneme, in which an intracellular increase in HCO_3_^−^ concentration was mediated by CO_2_ conversion (Inaba et al. 2003). Lastly, in the presence of sulphate ions (SO_4_^2−^), the rise in CO_3_^2−^ concentration in NAM adjusted at pH ≥ 7.4 could cause precipitation of Ca^2+^ in the form of CaCO_3_ (Mayorga et al. 2019), reducing Ca^2+^ bioavailability in NAM and inhibiting sperm motility. Biochemical analysis of our NAM did not show a difference in Ca^2+^ concentration at different pH levels (unpublished data). However, some precipitation during chilled storage was observed in the stock solutions that contained NaHCO_3_ buffers. The concentration of bioavailable Ca^2+^ may have differed between NAM adjusted at different pH levels during sperm storage, causing sperm motility to drop at a pH greater than 7.4. Therefore, further studies are required to elucidate the detailed molecular and ionic mechanism regulating sperm motility in barramundi.

Although the interaction of NaHCO_3_ with the sperm molecular pathway remains to be elucidated, in our study, we demonstrated that HEPES was a suitable substitute for NaHCO_3_ as a pH buffering agent. The use of the HEPES buffering agent permitted the investigation of the effect of pH on barramundi sperm motility after chilled storage. After 24 h incubation, sperm motility was significantly higher in HEPES-buffered NAM with pH 7.4 and 7.8 than in NAM at pH 6.5, 8.1, and 8.5. These results contrast slightly with what has been found in other species. For instance, in goldfish (*Carassius auratus*) and common carp (*Cyprinus carpio*), a significant decrease in sperm motility was observed when spermatozoa were stored in acidic (pH 6.5) but not alkaline conditions (pH 8.0 and 8.5) (Billard et al. 1995; Chantzaropoulos et al. 2015). These different responses could be due to the species-specific pH requirement of the mitochondrial ATP-synthase to function (Alavi and Cosson 2005; Vílchez et al. 2017). It was also noted that optimal sperm motility was found in either NAM adjusted at pH 7.4 or 7.8 and was consistent for each male barramundi over time. Changes in seminal plasma pH from 7.5 to 8.0 have been linked to the progressive maturation of spermatozoa in rainbow trout (*Oncorhynchus mykiss*) (Morisawa and Morisawa 1988) and Japanese eel (Billard et al. 1995; Ohta et al. 1997). In turn, variation in optimal NAM pH could indicate asynchronous sperm maturation amongst males, supporting previous observations of skewed paternity during mass-spawning (Frost et al. 2006; Robinson et al. 2010; Domingos et al. 2013, 2014, 2021; Loughnan et al. 2013; Marc et al. 2021; Guppy et al. 2022). Further studies should focus on (1) validating a mitochondrial membrane potential assay to monitor sperm mitochondrial function (e.g., JC-1 staining); and (2) characterizing sperm quality and seminal plasma characteristics of males at different gonadal maturation stages. Ultimately, this information will permit the refinement of pH in NAM and ensure optimal chilled storage of barramundi spermatozoa.

The results of the present study demonstrated the dependency of sperm motility on specific ions in barramundi for the first time. Barramundi sperm motility was inhibited within 1 h in Na^+^ free medium like in European eel (Vílchez et al. 2016), which contrasts with results in pufferfish (*Takifugu alboplumbeus*) where no decline in sperm motility was observed (Pérez et al. 2022). However, in contrast to the Japanese eel (Ohta et al. 2001) and the European eel (Vílchez et al. 2016, 2017), barramundi sperm motility improved at low K^+^ concentration (5 mM), similar to that in blood plasma (mean: 3.7 ± 0.1 mM), rather than at higher K^+^ concentration (30 mM), similar to that in seminal plasma. Although incubation in K^+^ free NAM did not deplete barramundi spermatozoa of their potential to become motile as it did in Japanese eel (Ohta et al. 1997), higher K^+^ concentration in NAM could alkalinize sperm intracellular pH, also affecting sperm mobility upon activation (Alavi et al. 2019). Other variables in seminal plasma, such as the Ca^2+^/K^+^ ratio, could also affect sperm motility performance, as demonstrated in Persian sturgeon (*Acipenser persicus*) (Alavi et al. 2006). Further research is needed to determine whether smaller increments of K^+^ between 5 and 30 mM in NAM could improve sperm motility further and how it affects sperm intracellular pH and ionic content during storage.

Overall, while the total motility achieved in the optimized NAM was substantially improved throughout this study from 20% initially to ~ 60%, it is lower than reported in many species (e.g., 90% in common carp (Cejko et al. 2018) and *Prochilodus lineatus* (Viveiros et al. 2016), ~ 80% in red seabream, *Pagrus major* (Liu et al. 2007)). Yet, it is concurrent with levels found in turbot (Suquet et al. 2000) and Japanese eel (Ohta et al. 1997) that used cannulation to collect spermatozoa. Therefore, further research is warranted to develop hormone-induced methods to strip spermatozoa for barramundi to understand better the influence of the collection method and sperm maturation on motility. Lastly, the development of hormone-induced final oocyte maturation will also enable the striping of eggs from females, thus testing the fertility potential of chilled spermatozoa.

## Conclusion

This study provides an optimized NAM for the short-term chilled storage of barramundi spermatozoa collected by testicular cannulation. We demonstrated that adjusting the osmolality to 400 mOsm/kg and pH to 7.4, in line with values found in seminal plasma, was optimal for maintaining sperm viability and motility. Furthermore, the replacement of NaHCO_3_ with HEPES as a pH buffer was able to overcome NaHCO_3_^−^-induced inhibition of sperm motility. Finally, Na^+^ at 185 mM and K^+^ at 5 mM in the medium retained the best sperm motility activation potential in captive-bred barramundi. These results lay the foundation for future studies looking at the regulatory role of different ions in barramundi sperm motility to further improve medium composition. More importantly, this optimized NAM facilitates safe handling and short-term storage of testicular spermatozoa, thereby facilitating the development of advanced reproductive technologies, including artificial fertilization programs, for barramundi.

## Supplementary Information

Below is the link to the electronic supplementary material.

Supplementary file1 (DOCX 244 KB)

## Data Availability

All data generated or analyzed during this study are included in this published article and its supplementary information files.
