# Supplementary material for: Optimization of a non-activating medium for short-term chilled storage of barramundi (Lates calcarifer) testicular spermatozoa
Source: Fish Physiol Biochem. 2023 May 17;49(4):559–76. doi: 10.1007/s10695-023-01191-8 (PMC10415525; doi:10.1007/s10695-023-01191-8)
Supplement: Supplementary file 1 — Supplementary file1 (DOCX 244 KB) [file 10695_2023_1191_MOESM1_ESM.docx]

**Supplementary Materials: Optimization of a non-activating medium for short-term chilled storage of barramundi (*Lates calcarifer*) testicular spermatozoa.**

Adrien F. Marc^abc*^, Jarrod L. Guppy^cd^, Hayley Marshall^b^, Dean R. Jerry^cdf^, Donna Rudd^be1^, and Damien B.B.P. Paris^ac1^

^a^ Gamete and Embryology (GAME) Laboratory, College of Public Health, Medical & Veterinary Sciences, James Cook University, Townsville, QLD 4811, Australia

^b^ College of Public Health, Medical, and Veterinary Sciences, James Cook University, Townsville, QLD 4811, Australia

^c^ Centre for Sustainable Tropical Fisheries and Aquaculture, College of Science and Engineering, James Cook University, Townsville, QLD 4811, Australia

^d^ Australian Research Council Industrial Transformation Research Hub for Advanced Prawn Breeding, James Cook University, Townsville, QLD 4811, Australia

^e^ Australian Institute of Tropical Health and Medicine, James Cook University, Townsville, QLD 4811, Australia

^f^ Tropical Futures Institute, James Cook University, Geylang, Singapore

^1^The work was jointly conducted in the laboratories of these two senior authors

**Corresponding author:**

Adrien F. Marc, James Cook University, Townsville, QLD 4811, Australia.

adrien.marc@my.jcu.edu.au; ORCID: 0000-0002-9364-6438

*Supplementary Material 1:* Effect of NaHCO_3_-buffered NAM sodium and potassium concentration on sperm motility.

**Methods:**

After determining the optimal NAM osmolality, the ratio of NaCl and KCl was assessed to determine whether a ratio of Na^+^/K^+^ ions similar to barramundi seminal plasma would improve sperm motility after saltwater activation. Different NaHCO_3_-buffered NAM treatments were prepared with the following NaCl/KCl concentrations: 0 mM NaCl/190 mM KCl, 140 mM NaCl/50 mM KCl, 160 mM NaCl/30 mM KCl, 170 mM NaCl/20 mM KCl, 190 mM NaCl/0 mM KCl. All media were maintained to an osmolality of 400 mOsm/kg and adjusted to pH 7.4 with 0.1 M NaOH. Sperm samples from *n* = 10 males were stored undiluted (control) or diluted at 1:10 in the different NAM treatments and incubated at 4 ℃ for 1 h and 24 h before motility was assessed by CASA.

**Results:**

After 1 h incubation, barramundi sperm motility did not differ across treatments (Supplementary Fig. S1; Supplementary Table S1). Overall, sperm motility was low, with the highest motility observed when stored undiluted (TM: 32.0 ± 4.8%). After 24 h incubation, sperm motility significantly declined for all treatments except 160 mM NaCl/30 mM KCl (Supplementary Fig. S1). However, sperm motility was highest, yet still very low, when stored undiluted (TM: 11.6 ± 2.6%). Spermatozoa incubated in 0 mM NaCl/190 mM KCl showed significantly lower sperm motility (PM, Medium, and Fast) and velocities (VCL, VSL, VAP, LIN, WOB, and BCF; Supplementary Table S1) than the control. However, except for the 0 mM/190 mM KCl medium, sperm motility and trajectory did not differ between treatments (Supplementary Table S1).


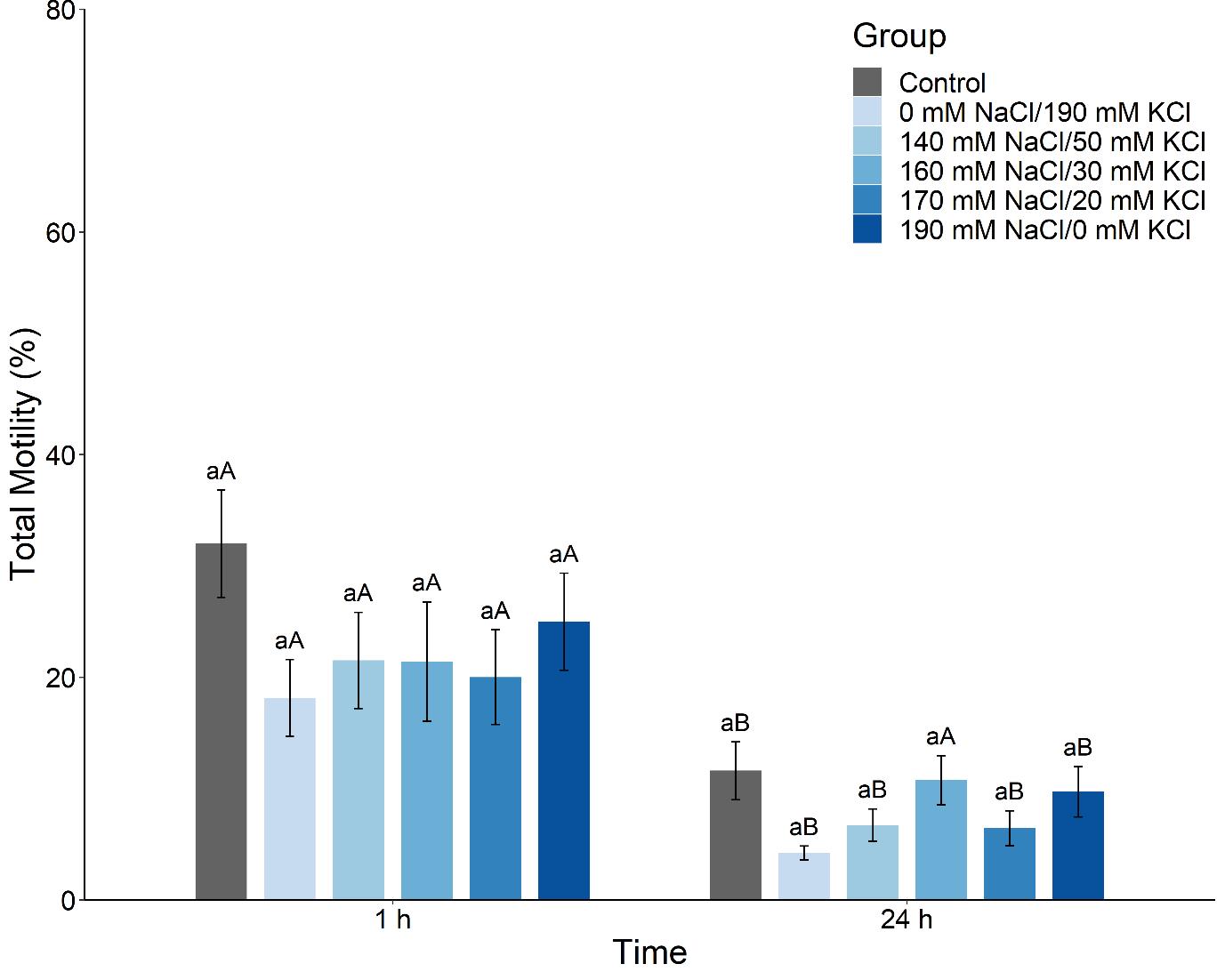


*Supplementary Fig. S1:* Effect of NaHCO_3_-buffered non-activating medium (NAM) NaCl and KCl concentration on sperm motility. Total motility of barramundi (*Lates calcarifer*) spermatozoa was analyzed at 1 and 24 h post-incubation at 4 °C in NaHCO_3_-buffered NAM with different NaCl and KCl concentrations (*n* = 10). Undiluted sperm was used as a control. NaCl and KCl concentrations of NAM (Marine Ringers’solution consisted of 0–190 mM NaCl, 0–190 mM KCl, 1.6 mM CaCl_2_·2H_2_O, 1.1 mM MgSO_4_·7H_2_O, 0.1 mM NaHCO_3_, 2.6 mM NaH_2_PO_4_. 2H_2_O and 5.6 mM D^+^ glucose at pH 7.4 and osmolality 400 mOsm/kg) were modified accordingly to maintain osmolality of 400 mOsm/kg. Data are mean ± SEM. Different lowercase letters indicate a significant difference between concentrations within an incubation period, and different capital letters indicate a significant difference between incubation periods at the same concentration (*P* < 0.05).

*Supplementary Table S1:* Effect of NaHCO_3_-buffered non-activating medium (NAM) NaCl and KCl concentration on sperm motility. Motility parameters of barramundi (*Lates calcarifer)* spermatozoa were analyzed at 1 and 24 h post-incubation at 4 °C in NaHCO_3_-buffered NAM with different NaCl and KCl concentrations (*n* = 10). Undiluted sperm was used as a control. NaCl and KCl concentrations of NAM (Marine Ringers’solution consisted of 0–190 mM NaCl, 0–190 mM KCl, 1.6 mM CaCl_2_·2H_2_O, 1.1 mM MgSO_4_·7H_2_O, 0.1 mM NaHCO_3_, 2.6 mM NaH_2_PO_4_. 2H_2_O and 5.6 mM D^+^ glucose at pH 7.4 and osmolality 400 mOsm/kg) were modify accordingly to maintain osmolality of 400 mOsm/kg.

|  | **1 h incubation** | |  |  |  |  |  | **24 h incubation** | |  |  |  |  |  |
| --- | --- | --- | --- | --- | --- | --- | --- | --- | --- | --- | --- | --- | --- | --- |
| **Parameter** | **Control** | **0 mM NaCl/**  **190 mM KCl** | **140 mM NaCl/**  **50 mM KCl** | **160 mM NaCl/**  **30 mM KCl** | **170 mM NaCl/**  **20 mM KCl** | **190 mM NaCl/**  **0 mM KCl** |  | **Control** | **0 mM NaCl/**  **190 mM KCl** | **140 mM NaCl/**  **50 mM KCl** | **160 mM NaCl/**  **30 mM KCl** | **170 mM NaCl/**  **20 mM KCl** | **190 mM NaCl/**  **0 mM KCl** |  |
| TM (%) | 32.0 ± 4.8^aA^ | 18.1 ± 3.5^aA^ | 21.5 ± 4.3^aA^ | 21.4 ± 5.4^aA^ | 20.0 ± 4.3^aA^ | 25.0 ± 4.4^aA^ |  | 11.6 ± 2.6^aB^ | 4.2 ± 0.6^aB^ | 6.7 ± 1.5^aB^ | 10.8 ± 2.2^aA^ | 6.5 ± 1.6^aB^ | 9.7 ± 2.3^aB^ |  |
| PM (%) | 18.5 ± 3.2^aA^ | 8.1 ± 2.1^aA^ | 10.2 ± 2.9^aA^ | 10.7 ± 3.0^aA^ | 8.4 ± 2.2^aA^ | 12.1 ± 2.9^aA^ |  | 5.4 ± 1.8^aB^ | 0.5 ± 0.2^bB^ | 2.0 ± 0.8^abB^ | 3.5 ± 1.1^abB^ | 1.8 ± 0.8^abB^ | 3.1 ± 1.0^abB^ |  |
| Slow (%) | 13.5 ± 2.3^aA^ | 10.1 ± 1.8^aA^ | 11.3 ± 1.9^aA^ | 10.7 ± 2.3^aA^ | 11.6 ± 2.5^aA^ | 12.9 ± 1.7^aA^ |  | 6.2 ± 1.1^aB^ | 3.7 ± 0.5^aB^ | 4.7 ± 0.8^aB^ | 7.3 ± 1.3^aA^ | 4.6 ± 0.8^aB^ | 6.6 ± 1.4^aB^ |  |
| Medium (%) | 11.6 ± 1.6^aA^ | 5.1 ± 1.3^bA^ | 6.1 ± 1.7^abA^ | 6.6 ± 1.6^abA^ | 5.5 ± 1.4^abA^ | 8.4 ± 1.9^abA^ |  | 3.9 ± 1.3^aB^ | 0.4 ± 0.1^bB^ | 1.3 ± 0.6^abB^ | 2.7 ± 0.8^abB^ | 1.4 ± 0.6^abB^ | 2.5 ± 0.9^abB^ |  |
| Fast (%) | 6.8 ± 1.9^aA^ | 3.0 ± 1.1^aA^ | 4.2 ± 1.5^aA^ | 4.1 ± 1.9^aA^ | 2.9 ± 1.0^aA^ | 3.7 ± 1.2^aA^ |  | 1.5 ± 0.7^aB^ | 0.1 ± 0.1^bB^ | 0.7 ± 0.3^abB^ | 0.8 ± 0.4^abB^ | 0.4 ± 0.2^abB^ | 0.7 ± 0.3^aB^ |  |
| VCL (µm/s) | 63.8 ± 6.7^aA^ | 48.2 ± 8.3^aA^ | 54.3 ± 10.8^aA^ | 51.5 ± 8.3^aA^ | 49.4 ± 9.5^aA^ | 52.8 ± 6.4^aA^ |  | 42.9 ± 6.4^aB^ | 22.7 ± 1.6^bB^ | 32.3 ± 3.9^abB^ | 33.8 ± 4.1^aB^ | 29.6 ± 3.4^abB^ | 38.1 ± 5.1^aB^ |  |
| VSL (µm/s) | 39.2 ± 5.5^aA^ | 29.0 ± 7.8^aA^ | 33.6 ± 10.4^aA^ | 29.4 ± 6.9^aA^ | 28.3 ± 8.6^aA^ | 27.5 ± 4.9^aA^ |  | 20.0 ± 4.9^aB^ | 3.1 ± 1.0^bB^ | 10.4 ± 3.3^aB^ | 13.1 ± 4.4^aB^ | 8.9 ± 2.9^abB^ | 12.2 ± 3.1^aA^ |  |
| VAP (µm/s) | 52.3 ± 7.1^aA^ | 34.7 ± 8.6^aA^ | 41.9 ± 11.7^aA^ | 39.5 ± 8.8^aA^ | 36.5 ± 10.0^aA^ | 39.8 ± 6.9^aA^ |  | 30.8 ± 7.0^aB^ | 7.3 ± 1.6^bB^ | 17.3 ± 4.5^abB^ | 19.0 ± 4.8^abB^ | 14.6 ± 3.8^abB^ | 21.9 ± 4.9^aB^ |  |
| LIN (%) | 48.8 ± 2.8^aA^ | 36.5 ± 4.7^aA^ | 39.8 ± 3.9^aA^ | 41.5 ± 4.3^aA^ | 36.1 ± 4.2^aA^ | 41.1 ± 3.4^aA^ |  | 29.9 ± 5^aB^ | 11.8 ± 1.9^bB^ | 19.1 ± 3.9^abB^ | 25.5 ± 5.2^aB^ | 19 ± 4.7^abB^ | 21.6 ± 4.1^abB^ |  |
| STR (%) | 66.6 ± 2.6^aA^ | 59.9 ± 3.3^aA^ | 62.2 ± 2.5^aA^ | 62.4 ± 3.7^aA^ | 57.8 ± 2.7^aA^ | 61.7 ± 2.8^aA^ |  | 49 ± 3.8^abB^ | 34.8 ± 3.2^bB^ | 40.4 ± 3.8^abB^ | 52.9 ± 4.1^abB^ | 42.2 ± 4.9^abB^ | 44.3 ± 4.2^abB^ |  |
| WOB (%) | 66.2 ± 2.4^aA^ | 49.7 ± 4.4^bA^ | 54.6 ± 4.2^bA^ | 58.3 ± 4^abA^ | 52.2 ± 4.3^bA^ | 58.7 ± 3.9^abA^ |  | 50.7 ± 6^aB^ | 28.4 ± 2^bB^ | 36.8 ± 4.3^abB^ | 40.8 ± 5.1^abB^ | 36.3 ± 4.8^abB^ | 41.7 ± 4.1^abB^ |  |
| ALH (µm) | 0.47 ± 0.01^aA^ | 0.44 ± 0.02^aA^ | 0.44 ± 0.01^aA^ | 0.47 ± 0.01^aA^ | 0.45 ± 0.01^aA^ | 0.47 ± 0.01^aA^ |  | 0.42 ± 0.03^aB^ | 0.40 ± 0.02^aA^ | 0.41 ± 0.01^aA^ | 0.43 ± 0.01^aB^ | 0.41 ± 0.01^aB^ | 0.46 ± 0.03^aA^ |  |
| BCF [Hz] | 11.3 ± 0.8^aA^ | 8.2 ± 1.1^bA^ | 8.8 ± 0.9^abA^ | 8.8 ± 1.0^abA^ | 7.9 ± 0.8^bA^ | 9.7 ± 0.8^abA^ |  | 7.2 ± 1.2^aB^ | 3.3 ± 0.6^aB^ | 5.6 ± 1.1^aB^ | 6.3 ± 1.0^aB^ | 5.4 ± 1.2^aA^ | 6.5 ± 0.9^aB^ |  |
| Data are mean ± standard error. Total motility (TM; VCL ≥ 15 µm/s); Progressive motility (PM; VCL ≥ 35 µm/s); Slow motility (VCL ≥ 15 and ˂ 35 µm/s), Medium motility (VCL ≥ 35 and ˂ 100 µm/s), Fast motility (VCL ≥ 100 µm/s). VCL, curvilinear velocity; VSL, straight-line velocity; VAP, average path velocity; LIN, linearity; STR, straightness; WOB, wobble; ALH, amplitude of lateral head displacement; BCF, beat cross frequency. Different lowercase letters indicate a significant difference between concentrations within an incubation period, and different capital letters indicate a significant difference between incubation periods at the same concentration (*P* < 0.05). | | | | | | | | | | | | | | |

*Supplementary Table S2:* Effect of NaHCO_3_-buffered non-activating medium (NAM) pH on sperm motility. Motility parameters of barramundi (*Lates calcarifer*) spermatozoa were analyzed at 1 and 24 h post-incubation at 4 °C in NaHCO_3_-buffered NAM with different pH (*n* = 10). Undiluted sperm was used as a control. pH of NAM (Marine Ringer's solution consisted of 182.4 mM NaCl, 5.1 mM KCl, 1.6 mM CaCl_2_·2H_2_O, 1.1 mM MgSO_4_·7H_2_O, 0.1 mM NaHCO_3_, 2.6 mM NaH_2_PO_4_·2H_2_O, and 5.6 mM D^+^ glucose, osmolality 400 mOsm/kg) was modified using NaOH.

|  | **1 h incubation** | |  |  |  |  |  | **24 h incubation** | |  |  |  |  |  |
| --- | --- | --- | --- | --- | --- | --- | --- | --- | --- | --- | --- | --- | --- | --- |
| **Parameter** | **Control** | **pH 6.5** | **pH 7.4** | **pH 7.8** | **pH 8.1** | **pH 8.5** |  | **Control** | **pH 6.5** | **pH 7.4** | **pH 7.8** | **pH 8.1** | **pH 8.5** |  |
| TM (%) | 41.8 ± 3.8^aA^ | 33.4 ± 3.0^abA^ | 23.2 ± 2.0^bcA^ | 24.2 ± 2.8^bcA^ | 17.4 ± 2.8^cA^ | 17.2 ± 3.3^cA^ |  | 17.2 ± 2.1^aB^ | 11.2 ± 2.4^aB^ | 1.8 ± 0.5^bB^ | 1.3 ± 0.2^bB^ | 1.8 ± 0.4^bB^ | 1.3 ± 0.2^bB^ |  |
| PM (%) | 26.8 ± 3.3^aA^ | 21.7 ± 2.5^abA^ | 13.9 ± 1.5^bcA^ | 15.7 ± 2.1^abcA^ | 10.9 ± 2.1^cA^ | 10.4 ± 2.5^cA^ |  | 8.8 ± 1.4^aB^ | 6.1 ± 1.5^aB^ | 0.2 ± 0.2^bB^ | 0.1 ± 0.0^bB^ | 0.1 ± 0.1^bB^ | 0.2 ± 0.1^bB^ |  |
| Slow (%) | 14.9 ± 1.6^aA^ | 11.8 ± 1.0^abA^ | 9.3 ± 0.9^bcA^ | 8.5 ± 0.8^bcA^ | 6.5 ± 0.8^cA^ | 6.8 ± 1.0^cA^ |  | 8.4 ± 1.0^aB^ | 5.1 ± 1.0^bB^ | 1.6 ± 0.3^cB^ | 1.2 ± 0.2^cB^ | 1.7 ± 0.4^cB^ | 1.1 ± 0.2^cB^ |  |
| Medium (%) | 12.5 ± 1.4^aA^ | 7.6 ± 0.9^abA^ | 5.7 ± 0.8^bA^ | 5.5 ± 0.5^bA^ | 4.2 ± 0.8^bA^ | 4.4 ± 1.0^bA^ |  | 3.8 ± 0.6^aB^ | 3.5 ± 0.9^aB^ | 0.2 ± 0.1^bB^ | 0.1 ± 0.0^bB^ | 0.1 ± 0.1^bB^ | 0.2 ± 0.1^bB^ |  |
| Fast (%) | 14.3 ± 3.1^aA^ | 14.0 ± 2.3^aA^ | 8.1 ± 1.1^abA^ | 10.2 ± 1.9^abA^ | 6.7 ± 1.4^abA^ | 5.9 ± 1.6^bA^ |  | 4.9 ± 0.9^aB^ | 2.6 ± 1.0^bB^ | 0.1 ± 0.1^cB^ | 0.0 ± 0.0^cB^ | 0.0 ± 0.0^cB^ | 0.0 ± 0.0^cB^ |  |
| VCL (µm/s) | 89.3 ± 9.4^aA^ | 111.3 ± 11.7^aA^ | 95.9 ± 7.2^aA^ | 101.1 ± 7.0^aA^ | 95.0 ± 9.3^aA^ | 88.0 ± 9.5^aA^ |  | 77.9 ± 7.2^aA^ | 65.1 ± 10.9^aB^ | 22.2 ± 1.8^bB^ | 21.2 ± 1.1^bB^ | 20.7 ± 1.0^bB^ | 20.2 ± 1.1^bB^ |  |
| VSL (µm/s) | 37.6 ± 5.4^aA^ | 52.7 ± 6.7^aA^ | 52.9 ± 7.2^aA^ | 49.4 ± 4.7^aA^ | 51.9 ± 8.0^aA^ | 46.9 ± 8.6^aA^ |  | 40.1 ± 7.1^aA^ | 24.4 ± 9.2^aB^ | 3.7 ± 0.9^bB^ | 3.1 ± 0.3^bB^ | 2.1 ± 0.3^cB^ | 2.6 ± 0.2^bcB^ |  |
| VAP (µm/s) | 76.2 ± 9.5^aA^ | 101.1 ± 11.3^aA^ | 87.2 ± 7.1^aA^ | 91.7 ± 7.0^aA^ | 87.1 ± 9.3^aA^ | 78.2 ± 9.7^aA^ |  | 68.3 ± 7.6^aA^ | 50.5 ± 11.6^aB^ | 8.9 ± 2.0^bB^ | 7.5 ± 0.5^bB^ | 6.5 ± 0.3^bB^ | 7.3 ± 0.6^bB^ |  |
| LIN (%) | 39.9 ± 1.6^bA^ | 47.4 ± 2.1^abA^ | 49.8 ± 1.7^aA^ | 47.7 ± 1.9^abA^ | 49.5 ± 2.3^abA^ | 46.7 ± 3.7^abA^ |  | 42.3 ± 2.6^aA^ | 31.5 ± 3.1^aB^ | 16.5 ± 2.8^bB^ | 15.4 ± 2^bB^ | 10.2 ± 1.4^cB^ | 13.8 ± 1.5^bcB^ |  |
| STR (%) | 54.8 ± 1.7^aA^ | 59.2 ± 2.5^aA^ | 62.3 ± 1.7^aA^ | 59.7 ± 1.9^aA^ | 61.3 ± 2.1^aA^ | 61.9 ± 3.4^aA^ |  | 57.5 ± 2.6^aA^ | 51.8 ± 1.9^abB^ | 43 ± 4.6^bcB^ | 39.1 ± 2.7^bcB^ | 30.3 ± 3.6^cB^ | 35.2 ± 2.7^cB^ |  |
| WOB (%) | 70.8 ± 2.3^aA^ | 77.9 ± 1.5^aA^ | 76.7 ± 1.2^aA^ | 77.5 ± 1.2^aA^ | 77.2 ± 1.5^aA^ | 72 ± 2.7^aA^ |  | 69.5 ± 1.8^aA^ | 59.6 ± 3.3^aB^ | 35.4 ± 3.1^bB^ | 36.3 ± 2.3^bB^ | 32.4 ± 2^bB^ | 37.6 ± 4.2^bB^ |  |
| ALH (µm) | 0.61 ± 0.03^aA^ | 0.65 ± 0.05^aA^ | 0.55 ± 0.02^abA^ | 0.60 ± 0.03^aA^ | 0.53 ± 0.03^bA^ | 0.55 ± 0.04^bA^ |  | 0.52 ± 0.02^aB^ | 0.56 ± 0.02^aA^ | 0.40 ± 0.02^bB^ | 0.40 ± 0.02^bB^ | 0.40 ± 0.02^bB^ | 0.41 ± 0.02^bB^ |  |
| BCF [Hz] | 9.8 ± 0.5^aA^ | 8.8 ± 0.6^aA^ | 9.4 ± 0.4^aA^ | 9.0 ± 0.4^aA^ | 9.1 ± 0.6^aA^ | 8.7 ± 0.6^aA^ |  | 8.0 ± 0.4^aB^ | 9.2 ± 0.7^aA^ | 3.9 ± 0.6^bB^ | 3.3 ± 0.5^bB^ | 2.9 ± 0.5^bB^ | 3.0 ± 0.4^bB^ |  |
| Data are mean ± standard error. Total motility (TM; VCL ≥ 15 µm/sec); Progressive motility (PM; VCL ≥ 35 µm/s); Slow motility (VCL ≥ 15 and ˂ 35 µm/s), Medium motility (VCL ≥ 35 and ˂ 100 µm/s), Fast motility (VCL ≥ 100 µm/s). VCL, curvilinear velocity; VSL, straight-line velocity; VAP, average path velocity; LIN, linearity; STR, straightness; WOB, wobble; ALH, amplitude of lateral head displacement; BCF, beat cross frequency. Different lowercase letters indicate a significant difference between pH treatments within an incubation period, and different capital letters indicate a significant difference between different incubation periods at the same pH (*P* < 0.05). | | | | | | | | | | | | | | |

|  | **1 h incubation** | |  |  |  |  |  |  | **24 h incubation** | |  |  |  |  |  |
| --- | --- | --- | --- | --- | --- | --- | --- | --- | --- | --- | --- | --- | --- | --- | --- |
| **Parameter** | **Control** | **pH 6.5B** | **pH 6.5H** | **pH 7.4** | **pH 7.8** | **pH 8.1** | **pH 8.5** |  | **Control** | **pH 6.5B** | **pH 6.5H** | **pH 7.4** | **pH 7.8** | **pH 8.1** | **pH 8.5** |
| TM (%) | 49.6 ± 2.7^aA^ | 50.7 ± 6.8^aA^ | 48.7 ± 5.3^aA^ | 51.8 ± 7.5^aA^ | 55.8 ± 4.0^aA^ | 45.9 ± 4.7^aA^ | 49.2 ± 3.5^aA^ |  | 28.1 ± 6.3^aB^ | 7.1 ± 1.2^bB^ | 9.3 ± 2.9^bB^ | 35.1 ± 3.3^aB^ | 29.7 ± 3.4^aB^ | 8.1 ± 2.4^bB^ | 8.4 ± 2.6^bB^ |
| PM (%) | 22.6 ± 2.7^aA^ | 28.8 ± 6.6^aA^ | 25.9 ± 3.3^aA^ | 31.1 ± 7.4^aA^ | 29.8 ± 3.7^aA^ | 24.9 ± 6.0^aA^ | 24.9 ± 4.1^aA^ |  | 10.4 ± 3.5^aA^ | 1.7 ± 0.9^bB^ | 3.3 ± 1.3^bB^ | 18.8 ± 3.0^aA^ | 13.0 ± 2.5^aB^ | 1.2 ± 0.6^bB^ | 2.1 ± 1.1^bB^ |
| Slow (%) | 27.0 ± 1.4^aA^ | 21.9 ± 1.0^aA^ | 22.9 ± 2.7^aA^ | 20.7 ± 1.7^aA^ | 26.1 ± 3.1^aA^ | 20.9 ± 2.6^aA^ | 24.4 ± 2.6^aA^ |  | 17.7 ± 2.9^aB^ | 5.3 ± 0.7^bB^ | 5.9 ± 1.7^bB^ | 16.2 ± 2.1^aA^ | 16.7 ± 2.3^aA^ | 6.9 ± 1.8^bB^ | 6.3 ± 1.5^bB^ |
| Medium (%) | 3.5 ± 0.4^aA^ | 1.9 ± 0.3^aA^ | 2.4 ± 0.6^aA^ | 2.3 ± 0.4^aA^ | 2.7 ± 0.3^aA^ | 1.7 ± 0.5^aA^ | 1.9 ± 0.2^aA^ |  | 1.8 ± 0.6^abA^ | 0.5 ± 0.2^bcB^ | 0.8 ± 0.2^abcB^ | 2.1 ± 0.2^aA^ | 1.1 ± 0.3^abcA^ | 0.2 ± 0.1^cA^ | 0.4 ± 0.2^bcA^ |
| Fast (%) | 19.1 ± 2.7^aA^ | 26.8 ± 6.3^aA^ | 23.5 ± 2.9^aA^ | 28.9 ± 7.3^aA^ | 27.1 ± 3.8^aA^ | 23.2 ± 6.2^aA^ | 23.0 ± 4.1^aA^ |  | 8.6 ± 3.0^aA^ | 1.2 ± 0.9^bB^ | 2.6 ± 1.1^bB^ | 16.8 ± 2.9^aA^ | 11.8 ± 2.5^aA^ | 1.0 ± 0.5^bB^ | 1.7 ± 1.1^bB^ |
| VCL (µm/s) | 81.5 ± 7.8^aA^ | 98.6 ± 8.7^aA^ | 102.5 ± 3.7^aA^ | 110.0 ± 10.5^aA^ | 108.0 ± 7.9^aA^ | 102.3 ± 15.5^aA^ | 105.8 ± 8.6^aA^ |  | 64.9 ± 6.6^abA^ | 36.9 ± 9.1^cB^ | 45.9 ± 8.2^bcB^ | 86.0 ± 8.6^aA^ | 83.2 ± 9.2^aA^ | 34.6 ± 6.1^cB^ | 35.1 ± 8.2^cB^ |
| VSL (µm/s) | 49.4 ± 7.1^aA^ | 46.8 ± 6.5^aA^ | 50.2 ± 7.1^aA^ | 65.1 ± 5.7^aA^ | 61.0 ± 3.7^aA^ | 49.2 ± 9.6^aA^ | 64.3 ± 7.1^aA^ |  | 33.5 ± 6.2^abcA^ | 9.7 ± 2.6^cB^ | 18.7 ± 7.0^abcA^ | 38.1 ± 6.4^abB^ | 43.1 ± 7.8^aA^ | 12.1 ± 4.0^bcB^ | 13.7 ± 6.7^bcB^ |
| VAP (µm/s) | 71.5 ± 8.6^aA^ | 87.5 ± 8.3^aA^ | 92.4 ± 4.1^aA^ | 100.5 ± 10.0^aA^ | 97.9 ± 7.4^aA^ | 88.8 ± 15.4^aA^ | 93.9 ± 8.7^aA^ |  | 53.9 ± 7.5^abA^ | 23.8 ± 9.5^cB^ | 33.4 ± 9.0^bcB^ | 74.7 ± 9.1^aA^ | 72.0 ± 9.5^aA^ | 22.4 ± 6.3^cB^ | 22.8 ± 9.0^cB^ |
| LIN (%) | 44.1 ± 3.7^aA^ | 39.1 ± 2.9^aA^ | 40.5 ± 4.1^aA^ | 47.2 ± 3.5^aA^ | 45.2 ± 1.3^aA^ | 37 ± 2.5^aA^ | 43.2 ± 3.8^aA^ |  | 38.3 ± 3.9^aA^ | 21 ± 2.8^bcB^ | 24.4 ± 2.4^abcB^ | 34.9 ± 3.6^abcB^ | 36.9 ± 3.4^abB^ | 24.6 ± 4.9^abcA^ | 20 ± 4.6^cA^ |
| STR (%) | 60.3 ± 3^aA^ | 51.5 ± 3^aA^ | 53.1 ± 4.6^aA^ | 59.2 ± 3.5^aA^ | 58.4 ± 1.7^aA^ | 51.3 ± 2^aA^ | 56.6 ± 3^aA^ |  | 56.6 ± 3.2^aA^ | 43.8 ± 2.2^aB^ | 43.5 ± 3.3^aA^ | 48.2 ± 3^aB^ | 52.6 ± 2.6^aA^ | 48.5 ± 6.3^aA^ | 39.5 ± 5.3^aA^ |
| WOB (%) | 67.8 ± 2.8^aA^ | 72 ± 2.4^aA^ | 73 ± 2^aA^ | 75.1 ± 2.3^aA^ | 72.9 ± 1.9^aA^ | 68.2 ± 3.6^aA^ | 68.9 ± 3.4^aA^ |  | 62.2 ± 3.1^abcA^ | 45.4 ± 6.4^bcA^ | 50.8 ± 4.6^abcB^ | 69.4 ± 3.8^aA^ | 64.6 ± 3.2^abA^ | 45.1 ± 4.9^bcB^ | 43.1 ± 5^cA^ |
| ALH (µm) | 0.51 ± 0.01^aA^ | 0.63 ± 0.04^aA^ | 0.62 ± 0.04^aA^ | 0.59 ± 0.04^aA^ | 0.59 ± 0.03^aA^ | 0.63 ± 0.04^aA^ | 0.60 ± 0.04^aA^ |  | 0.52 ± 0.02^abA^ | 0.46 ± 0.04^bcA^ | 0.45 ± 0.03^bcA^ | 0.56 ± 0.03^aA^ | 0.56 ± 0.02^aB^ | 0.45 ± 0.03^cB^ | 0.42 ± 0.02^cB^ |
| BCF [Hz] | 8.5 ± 0.4^aA^ | 6.7 ± 0.9^aA^ | 6.7 ± 0.6^aA^ | 8.5 ± 0.5^aA^ | 8.0 ± 0.6^aA^ | 8.5 ± 0.9^aA^ | 8.1 ± 1.0^aA^ |  | 6.6 ± 0.2^abB^ | 4.2 ± 0.4^bcB^ | 6.8 ± 0.9^aA^ | 8.3 ± 0.7^aA^ | 7.1 ± 0.6^aA^ | 3.7 ± 0.7^cB^ | 4.0 ± 0.4^cA^ |
| Data are mean ± standard error; Total motility (TM; VCL ≥ 15 µm/s); Progressive motility (PM; VCL ≥ 35 µm/s); Slow motility (VCL ≥ 15 and ˂ 35 µm/s), Medium motility (VCL ≥ 35 and ˂ 100 µm/s), Fast motility (VCL ≥ 100 µm/s). VCL, curvilinear velocity; VSL, straight-line velocity; VAP, average path velocity; LIN, linearity; STR, straightness; WOB, wobble; ALH, amplitude of lateral head displacement; BCF, beat cross frequency. Different lowercase letters indicate a significant difference between pH treatments within an incubation period, and different capital letters indicate a significant difference between different incubation periods at the same pH (*P* < 0.05). | | | | | | | | | | | | | | | |

*Supplementary Table S3:* Effect of HEPES-buffered non-activating medium (NAM) pH on sperm motility. Motility parameters of barramundi (*Lates calcarifer*) spermatozoa were analyzed at 1 and 24 h post-incubation at 4 °C in HEPES-buffered NAM with different pH (*n* = 7). Undiluted sperm was used as a control as well as sperm diluted in the NaHCO_3_-buffered NAM pH 6.5 for comparison. pH of NAM (Marine Ringer's solution consisted of 182.4 mM NaCl, 5.1 mM KCl, 1.6 mM CaCl_2_·2H_2_O, 1.1 mM MgSO_4_·7H_2_O, 10.0 mM HEPES, and 5.6 mM D^+^ glucose, osmolality 400 mOsm/kg) was modified using NaOH.

*Supplementary Table S4:* Effect of HEPES-buffered non-activating medium (NAM) NaCl and KCl concentration on sperm motility. Motility parameters of barramundi (*Lates calcarifer*) spermatozoa were analyzed at 1 and 24 h post-incubation at 4 °C in HEPES-buffered NAM with different NaCl and KCl concentrations (*n* = 6). Undiluted sperm was used as a control. NaCl and KCl concentrations of NAM (Marine Ringer's solution consisted of 0–190 mM NaCl, 0–190 mM KCl, 1.6 mM CaCl_2_·2H_2_O, 1.1 mM MgSO_4_·7H_2_O, 10.0 mM HEPES, and 5.6 mM D^+^ glucose at pH 7.4 and osmolality 400 mOsm/kg) were modified accordingly to maintain osmolality of 400 mOsm/kg.

|  | **1 h incubation** | |  |  |  |  |  | **24 h incubation** | |  |  |  |  |  |
| --- | --- | --- | --- | --- | --- | --- | --- | --- | --- | --- | --- | --- | --- | --- |
| **Parameter** | **Control** | **0 mM NaCl/**  **190 mM KCl** | **140 mM NaCl/**  **50 mM KCl** | **160 mM NaCl/**  **30 mM KCl** | **185 mM NaCl/**  **5 mM KCl** | **190 mM NaCl/**  **0 mM KCl** |  | **Control** | **0 mM NaCl/**  **190 mM KCl** | **140 mM NaCl/**  **50 mM KCl** | **160 mM NaCl/**  **30 mM KCl** | **185 mM NaCl/**  **5 mM KCl** | **190 mM NaCl/**  **0 mM KCl** |  |
| TM (%) | 43.5 ± 4.5^aA^ | 4.6 ± 0.6^bA^ | 47.1 ± 4.8^aA^ | 44.5 ± 3.5^aA^ | 56.8 ± 5.0^aA^ | 56.5 ± 4.3^aA^ |  | 24.8 ± 6.7^abcB^ | 8.8 ± 5.1^cA^ | 21.9 ± 5.3^bcB^ | 18.4 ± 3.4^bcB^ | 47.7 ± 7.2^aA^ | 27.7 ± 1.5^abB^ |  |
| PM (%) | 17.4 ± 2.3^aA^ | 0.4 ± 0.2^bA^ | 22.4 ± 3.1^aA^ | 21.1 ± 2.5^aA^ | 28.5 ± 2.4^aA^ | 25.3 ± 3.1^aA^ |  | 5.8 ± 1.9^abB^ | 3.8 ± 3.8^bA^ | 7.8 ± 3.1^abB^ | 6.0 ± 1.8^abB^ | 20.3 ± 3.6^aB^ | 10.4 ± 1.8^abB^ |  |
| Slow (%) | 26.1 ± 2.6^aA^ | 4.3 ± 0.6^bA^ | 24.8 ± 2.6^aA^ | 23.4 ± 1.7^aA^ | 28.3 ± 4.3^aA^ | 31.2 ± 1.9^aA^ |  | 19.0 ± 4.8^abA^ | 4.9 ± 1.4^cA^ | 14.0 ± 2.3^bcA^ | 12.4 ± 1.8^bcB^ | 27.3 ± 3.9^aA^ | 17.3 ± 0.6^abB^ |  |
| Medium (%) | 2.3 ± 0.4^aA^ | 0.2 ± 0.1^bA^ | 2.0 ± 0.6^aA^ | 2.3 ± 0.3^aA^ | 2.1 ± 0.3^aA^ | 2.8 ± 0.5^aA^ |  | 0.9 ± 0.4^abA^ | 0.1 ± 0.1^bA^ | 1.0 ± 0.5^abA^ | 1.6 ± 0.5^abA^ | 2.7 ± 0.5^aA^ | 1.7 ± 0.3^aA^ |  |
| Fast (%) | 15.1 ± 2.2^bA^ | 0.2 ± 0.2^cA^ | 20.4 ± 3.1^abcA^ | 18.8 ± 2.6^abA^ | 26.4 ± 2.3^aA^ | 22.5 ± 3.0^abA^ |  | 4.9 ± 1.6^abB^ | 3.7 ± 3.7^bA^ | 6.9 ± 2.6^abB^ | 4.4 ± 1.3^abB^ | 17.6 ± 3.3^aB^ | 8.7 ± 1.7^abB^ |  |
| VCL (µm/s) | 77.9 ± 6.8^bA^ | 25.4 ± 5.3^cA^ | 90.9 ± 8.1^abA^ | 95.6 ± 7.0^abA^ | 112.1 ± 7.4^aA^ | 99.3 ± 6.9^abA^ |  | 47.5 ± 6.1^bcB^ | 42.6 ± 20.8^cA^ | 54.8 ± 11.0^abcB^ | 52.1 ± 6.3^bcB^ | 78.8 ± 5.6^aB^ | 64.3 ± 4.8^abB^ |  |
| VSL (µm/s) | 48.2 ± 7.3^bA^ | 6.9 ± 4.4^cA^ | 61.4 ± 7.6^abA^ | 65.8 ± 6.6^abA^ | 79.1 ± 6.8^aA^ | 62.5 ± 6.8^abA^ |  | 26.4 ± 5.5^abcB^ | 20.8 ± 18.1^cA^ | 31.0 ± 8.0^abB^ | 26.8 ± 5.2^abcB^ | 42.7 ± 4.3^aB^ | 25.7 ± 2.5^bcB^ |  |
| VAP (µm/s) | 67.1 ± 7.0^bA^ | 12.1 ± 4.8^cA^ | 81.8 ± 8.4^abA^ | 86.5 ± 7.2^abA^ | 102.6 ± 7.9^aA^ | 87.9 ± 7.2^abA^ |  | 35.9 ± 6.5^bcB^ | 29.3 ± 21.8^cA^ | 45.5 ± 11.3^abB^ | 41.0 ± 6.8^bcB^ | 67.1 ± 5.8^aB^ | 50.5 ± 4.2^abB^ |  |
| LIN (%) | 41.5 ± 2.9^aA^ | 15.6 ± 3.3^bA^ | 48.2 ± 2.4^aA^ | 48 ± 2.6^aA^ | 50.1 ± 3.8^aA^ | 44.6 ± 3.7^aA^ |  | 31.6 ± 4.1^abA^ | 19.4 ± 8^bA^ | 37.1 ± 4.4^aB^ | 33.6 ± 3.5^aA^ | 38.5 ± 2.9^aB^ | 29 ± 2.3^abB^ |  |
| STR (%) | 59.2 ± 3^aA^ | 35.4 ± 5^bA^ | 63.1 ± 1.3^aA^ | 62.2 ± 2.5^aA^ | 63.6 ± 3.1^aA^ | 60.6 ± 3.7^aA^ |  | 51.9 ± 3.9^abA^ | 38.8 ± 6.3^bA^ | 55.8 ± 3.1^aB^ | 50.9 ± 2.9^abA^ | 55.1 ± 3.5^aB^ | 47.6 ± 2.9^abA^ |  |
| WOB (%) | 63.5 ± 1.2^aA^ | 38.7 ± 2.3^bA^ | 69.1 ± 2.2^aA^ | 69.8 ± 1.9^aA^ | 71.1 ± 3.1^aA^ | 67.1 ± 1.8^aA^ |  | 52.1 ± 3.3^bcB^ | 42.1 ± 7.8^cA^ | 59.5 ± 5^abB^ | 57.8 ± 3.4^abcA^ | 63.8 ± 2^aA^ | 57.3 ± 2.2^abcA^ |  |
| ALH (µm) | 0.51 ± 0.02^aA^ | 0.40 ± 0.01^bA^ | 0.50 ± 0.02^aA^ | 0.50 ± 0.01^aA^ | 0.55 ± 0.02^aA^ | 0.59 ± 0.04^aA^ |  | 0.45 ± 0.02^abB^ | 0.45 ± 0.03^abA^ | 0.43 ± 0.02^bB^ | 0.46 ± 0.02^abB^ | 0.56 ± 0.04^aA^ | 0.55 ± 0.03^abA^ |  |
| BCF [Hz] | 7.9 ± 0.5^abA^ | 3.3 ± 1.2^cA^ | 8.2 ± 0.6^abA^ | 8.8 ± 0.4^aA^ | 8.8 ± 0.8^abA^ | 7.4 ± 0.3^bcA^ |  | 5.4 ± 0.4^abB^ | 3.5 ± 1.2^bA^ | 6.7 ± 1.0^aA^ | 6.7 ± 0.7^aA^ | 6.8 ± 0.2^aA^ | 6.8 ± 0.5^aA^ |  |
| Data are mean ± standard error. Total motility (TM; VCL ≥ 15 µm/s); Progressive motility (PM; VCL ≥ 35 µm/s); Slow motility (VCL ≥ 15 and ˂ 35 µm/s), Medium motility (VCL ≥ 35 and ˂ 100 µm/s), Fast motility (VCL ≥ 100 µm/s). VCL, curvilinear velocity; VSL, straight-line velocity; VAP, average path velocity; LIN, linearity; STR, straightness; WOB, wobble; ALH, amplitude of lateral head displacement; BCF, beat cross frequency. Different lowercase letters indicate a significant difference between concentration treatments within an incubation period, and different capital letters indicate a significant difference between incubation periods at the same concentration treatment (*P* < 0.05). | | | | | | | | | | | | | | |

*Supplementary Table S5*: Evaluation of the optimized NAM post-short-term chilled storage on sperm motility. Motility parameters of barramundi, (*Lates calcarifer*) spermatozoa were analyzed for up to 96 h at 4 °C in an optimized HEPES-buffered non-activating medium (*n* = 6). Undiluted sperm was used as a control. Optimized NAM consisted of 185 mM NaCl, 5.0 mM KCl, 1.6 mM CaCl_2_·2H_2_O, 1.1 mM MgSO_4_·7H_2_O, 10 mM HEPES and 5.6 mM D^+^ glucose at pH 7.4 and osmolality 400 mOsm/kg.

|  | **1 h incubation** | |  | | **24 h incubation** | |  | **48 h incubation** | |  | **72 h incubation** | | |  | **96 h incubation** | |
| --- | --- | --- | --- | --- | --- | --- | --- | --- | --- | --- | --- | --- | --- | --- | --- | --- |
| **Parameter** | **Control** | **Optimized NAM** | |  | **Control** | **Optimized NAM** |  | **Control** | **Optimized**  **NAM** |  | **Control** | **Optimized**  **NAM** | |  | **Control** | **Optimized NAM** |
| TM (%) | 43.5 ± 4.5^bA^ | 56.8 ± 5.0^aA^ |  | | 24.8 ± 6.7^aAB^ | 47.7 ± 7.2^aA^ |  | 12.6 ± 4.8^aBC^ | 29.3 ± 9.3^aAB^ |  | 7.4 ± 2.8^aBC^ | | 16.9 ± 6.7^aB^ |  | 3.1 ± 0.7^aC^ | 3.1 ± 0.6^aB^ |
| PM (%) | 17.4 ± 2.3^bA^ | 28.5 ± 2.4^aA^ |  | | 5.8 ± 1.9^bB^ | 20.3 ± 3.6^aAB^ |  | 4.4 ± 2.6^aB^ | 9.8 ± 3.4^aBC^ |  | 2.2 ± 1.5^aB^ | | 5.7 ± 2.6^aC^ |  | 0.0 ± 0.0^aC^ | 0.2 ± 0.1^aC^ |
| Slow (%) | 26.1 ± 2.6^aA^ | 28.3 ± 4.3^aA^ |  | | 19.0 ± 4.8^aAB^ | 27.3 ± 3.9^aA^ |  | 8.2 ± 2.5^aBC^ | 19.5 ± 6.0^aAB^ |  | 5.2 ± 1.5^aBC^ | | 11.1 ± 4.3^aAB^ |  | 3.1 ± 0.7^aC^ | 2.9 ± 0.5^aB^ |
| Medium (%) | 2.3 ± 0.4^aA^ | 2.1 ± 0.3^aA^ |  | | 0.9 ± 0.4^bAB^ | 2.7 ± 0.5^aA^ |  | 0.4 ± 0.2^aB^ | 1.8 ± 0.6^aAB^ |  | 0.2 ± 0.1^aB^ | | 0.5 ± 0.3^aAB^ |  | 0.0 ± 0.0^aB^ | 0.2 ± 0.1^aB^ |
| Fast (%) | 15.1 ± 2.2^bA^ | 26.4 ± 2.3^aA^ |  | | 4.9 ± 1.6^bB^ | 17.6 ± 3.3^aB^ |  | 4.0 ± 2.6^aAB^ | 8.0 ± 3.0^aBC^ |  | 1.9 ± 1.6^aBC^ | | 5.2 ± 2.4^aC^ |  | 0.0 ± 0.0^aC^ | 0.0 ± 0.0^aC^ |
| VCL (µm/s) | 77.9 ± 6.8^bA^ | 112.1 ± 7.4^aA^ |  | | 47.5 ± 6.1^bB^ | 78.8 ± 5.6^aB^ |  | 60.8 ± 17.0^aAB^ | 56.3 ± 15.3^aBC^ |  | 38.5 ± 12.0^aAB^ | | 55.2 ± 12.9^aBC^ |  | 19.6 ± 0.9^aC^ | 20.1 ± 1.4^aC^ |
| VSL (µm/s) | 48.2 ± 7.3^bA^ | 79.1 ± 6.8^aA^ |  | | 26.4 ± 5.5^aA^ | 42.7 ± 4.3^aB^ |  | 32.7 ± 13.1^aAB^ | 35.1 ± 15.6^aABC^ |  | 14.8 ± 10.5^aAB^ | | 25.1 ± 10.0^aBC^ |  | 2.2 ± 0.9^aB^ | 1.9 ± 0.7^aC^ |
| VAP (µm/s) | 67.1 ± 7.0^bA^ | 102.6 ± 7.9^aA^ |  | | 35.9 ± 6.5^bB^ | 67.1 ± 5.8^aB^ |  | 49.1 ± 18.4^aABC^ | 44.4 ± 16.1^aBC^ |  | 24.4 ± 13.2^aABC^ | | 44.7 ± 13.4^aBC^ |  | 6.5 ± 1.1^aC^ | 6.1 ± 1.2^aC^ |
| LIN (%) | 41.5 ± 2.9^bA^ | 50.1 ± 3.8^aA^ |  | | 31.6 ± 4.1^aA^ | 38.5 ± 2.9^aA^ |  | 28.7 ± 7.2^aAB^ | 32.5 ± 9.1^aAB^ |  | 19.2 ± 6.2^aAB^ | | 27.4 ± 7.4^aAB^ |  | 10.1 ± 3.1^aB^ | 7.3 ± 1.5^aB^ |
| STR (%) | 59.2 ± 3^aA^ | 63.6 ± 3.1^aA^ |  | | 51.9 ± 3.9^aA^ | 55.1 ± 3.5^aA^ |  | 44.4 ± 7.5^aAB^ | 49.5 ± 10.1^aAB^ |  | 39.7 ± 4.4^aAB^ | | 44.5 ± 7.3^aAB^ |  | 26.8 ± 2.8^aB^ | 24.6 ± 2.8^aB^ |
| WOB (%) | 63.5 ± 1.2^bA^ | 71.1 ± 3.1^aA^ |  | | 52.1 ± 3.3^aB^ | 63.8 ± 2^aA^ |  | 51.1 ± 8^aABC^ | 52.3 ± 7.9^aAB^ |  | 41.4 ± 6.6^aABC^ | | 52.5 ± 8.4^aAB^ |  | 31.7 ± 4.1^aC^ | 27.6 ± 2.7^aB^ |
| ALH (µm) | 0.51 ± 0.02^aA^ | 0.55 ± 0.02^aA^ |  | | 0.45 ± 0.02^bA^ | 0.56 ± 0.04^aA^ |  | 0.46 ± 0.04^aAB^ | 0.51 ± 0.07^aAB^ |  | 0.44 ± 0.02^aAB^ | | 0.45 ± 0.04^aAB^ |  | 0.34 ± 0.03^aB^ | 0.36 ± 0.03^aB^ |
| BCF [Hz] | 7.9 ± 0.5^aA^ | 8.8 ± 0.8^aA^ |  | | 5.4 ± 0.4^aB^ | 6.8 ± 0.2^aA^ |  | 5.3 ± 1.2^aABC^ | 5.5 ± 1.5^aAB^ |  | 4.5 ± 0.8^aBC^ | | 4.6 ± 1.0^aB^ |  | 1.8 ± 0.4^aC^ | 1.9 ± 0.3^aB^ |
| Data are mean ± standard error. Total motility (TM; VCL ≥ 15 µm/s); Progressive motility (PM; VCL ≥ 35 µm/s); Slow motility (VCL ≥ 15 and ˂ 35 µm/s), Medium motility (VCL ≥ 35 and ˂ 100 µm/s), Fast motility (VCL ≥ 100 µm/s). VCL, curvilinear velocity; VSL, straight-line velocity; VAP, average path velocity; LIN, linearity; STR, straightness; WOB, wobble; ALH, amplitude of lateral head displacement; BCF, beat cross frequency. Different lowercase letters indicate a significant difference between extender treatments within an incubation period, and different capital letters indicate a significant difference between incubation periods for the same medium(*P* < 0.05). | | | | | | | | | | | | | | | | |

*Supplementary Table S6*: Individual variation of total motility of barramundi (*Lates calcarifer*) spermatozoa analyzed for up to 96 h at 4 °C in an optimized HEPES-buffered non-activating medium (*n* = 6). Undiluted sperm was used as a control. Optimized NAM consisted of 185 mM NaCl, 5.0 mM KCl, 1.6 mM CaCl_2_·2H_2_O, 1.1 mM MgSO_4_·7H_2_O, 10 mM HEPES and 5.6 mM D^+^ glucose at pH 7.4 and osmolality 400 mOsm/kg.

|  | **1 h incubation** | |  | **24 h incubation** | |  | **48 h incubation** | |  | **72 h incubation** | |  | **96 h incubation** | |
| --- | --- | --- | --- | --- | --- | --- | --- | --- | --- | --- | --- | --- | --- | --- |
| **Male** | **Control** | **Optimized NAM** |  | **Control** | **Optimized NAM** |  | **Control** | **Optimized NAM** |  | **Control** | **Optimized NAM** |  | **Control** | **Optimized NAM** |
| 1 | 40.1 ± 7.5 | 54.4 ± 2.4 |  | 10.3 ± 0.7 | 16.3 ± 1.6 |  | 3.2 ± 1.1 | 2.0 ± 0.8 |  | 2.1 ± 1.0 | 0.0 ± 0.0 |  | 1.9 ± 0.4 | 2.1 ± 0.7 |
| 2 | 41.9 ± 3.5 | 51.2 ± 4.7 |  | 18.6 ± 2.0 | 49.4 ± 5.4 |  | 3.5 ± 1.4 | 2.9 ± 1.4 |  | 3.7 ± 1.1 | 3.2 ± 1.2 |  | 6.2 ± 0.6 | 3.2 ± 1.1 |
| 3 | 46.6 ± 2.1 | 62.5 ± 2.1 |  | 34.4 ± 6.0 | 59.1 ± 2.2 |  | 9.7 ± 2.1 | 25.3 ± 3.2 |  | 1.7 ± 0.6 | 3.0 ± 2.2 |  | 1.0 ± 0.5 | 4.1 ± 0.8 |
| 4 | 61.7 ± 2.9 | 65.8 ± 3.2 |  | 48.1 ± 3.1 | 66.8 ± 0.3 |  | 19.1 ± 3.5 | 49.4 ± 5.9 |  | 11.5 ± 2.8 | 35.4 ± 3.5 |  | 3.8 ± 1.2 | 5.3 ± 0.8 |
| 5 | 43.2 ± 3.8 | 70.4 ± 2.4 |  | 32.7 ± 1.1 | 53.2 ± 2.5 |  | 6.4 ± 1.9 | 51.8 ± 4.1 |  | 6.4 ± 2.1 | 31.8 ± 2.5 |  | 2.7 ± 0.4 | 2.8 ± 0.3 |
| 6 | 27.9 ± 3.8 | 36.8 ± 6.0 |  | 4.6 ± 0.9 | 41.2 ± 1.2 |  | 33.5 ± 3.3 | 44.1 ± 1.1 |  | 19.1 ± 1.9 | 27.7 ± 5.2 |  | 2.8 ± 1.0 | 1.3 ± 1.3 |
| Mean | 43.5 ± 4.5 | 56.8 ± 5.0 |  | 24.8 ± 6.7 | 47.7 ± 7.2 |  | 12.6 ± 4.8 | 29.3 ± 9.3 |  | 7.4 ± 2.8 | 16.9 ± 6.7 |  | 3.1 ± 0.7 | 3.1 ± 0.6 |
| Data are mean ± standard error. Total motility (VCL ≥ 15 µm/s). | | | | | | | | | | | | | | |

*Supplementary Table S7*: Mineral and organic composition of blood plasma of captive barramundi (*Lates calcarifer*) broodstock reared in freshwater.

|  | **Blood Plasma** | |  |
| --- | --- | --- | --- |
| **Parameter** | **Mean ± SEM** | **Range** | **CV** |
| Na^+^ (mM) | 105.6 ± 3.0 (8) | 93.3 – 114.2 | 7.9 |
| K^+^ (mM) | 4.7 ± 0.3 (8) | 3.9 – 5.8 | 15.9 |
| Ca^2+^ (mM) | 2.2 ± 0.1 (8) | 1.8 – 2.8 | 16.3 |
| Mg^2+^ (mM) | 0.5 ± 0.1 (8) | 0.4 – 0.8 | 27.9 |
| Cl^-^ (mM) | 80.2 ± 4.7 (8) | 63.5 – 92.6 | 16.5 |
| PO_4_ (mM) | 1.4 ± 0.3 (8) | 0.7 – 2.7 | 58.0 |
| pCO_2_ (mmHg) | 8.2 ± 0.4 (4) | 7.0 – 8.8 | 10.0 |
| TP (g.L^-1^) | 38.5 ± 0.8 (4) | 36.2 – 40.1 | 4.3 |
| CHO (mM) | 3.0 ± 0.3 (4) | 2.1 – 3.6 | 21.2 |
| Glu (mM) | 2.2 ± 0.4 (7) | 0.92 – 3.36 | 45.5 |
| TG (mM) | 0.8 ± 0.1 (4) | 0.59 – 0.94 | 20.4 |
| Urea (mM) | 1.7 ± 0.1 (7) | 1.6 – 2.2 | 10.7 |
| pH | 7.7 ± 0.0 (8) | 7.5 – 7.9 | 1.6 |
| Osm (mOsm/kg) | 218.3 ± 3.9 (4) | 211 – 229 | 3.5 |
| Data are mean ± standard error (SEM), number of fish (*n*), and coefficient of variation (CV) are reported for each parameter. Abbreviations: Total protein (TP), cholesterol (CHO), glucose (Glu), triglycerides (TG), and osmolality (Osm). | | | |
